# Liver Injury Associated With Irregular Herbal Products: A Translational Case Series With Chemical Authentication and Causality Assessment Using the Updated RUCAM

**DOI:** 10.1155/crhe/5905774

**Published:** 2026-07-17

**Authors:** Ferdinando Lucas Góis, Rebeca Santos do Amaral de Souza, Raymundo Paraná, Vinícius Nunes, Genario Oliveira Santos Júnior, Ademir Evangelista do Vale

**Affiliations:** ^1^ Postgraduate Program in Pharmaceutical Assistance (PPGASFAR), Faculty of Pharmacy, Federal University of Bahia (UFBA), Salvador, Bahia, Brazil, ufba.br; ^2^ Department of Gastroenterology, Professor Edgard Santos University Hospital (HUPES), Federal University of Bahia (UFBA), Salvador, Bahia, Brazil, ufba.br

**Keywords:** adulteration, chemical authentication, dietary supplements, drug-induced liver injury, hepatotoxicity, herb-induced liver injury, translational pharmacovigilance, updated RUCAM

## Abstract

**Background:**

Herb‐induced liver injury (HILI) represents an increasing diagnostic challenge owing to the widespread use of herbal and dietary supplements (HDS), which are frequently consumed without reliable information regarding their composition or safety. Mischaracterized products may obscure causal attributes and mimic primary herbal hepatotoxicity.

**Methods and Results:**

We report a case series of five patients with suspected HILI at a tertiary university hospital in Bahia, Brazil. Clinical causality was prospectively assessed using the updated Roussel Uclaf Causality Assessment Method (RUCAM, 2016). Most patients present with hepatocellular injury following exposure to commercially available HDS, frequently in the presence of metabolic comorbidities. Analytical authentication of the consumed products revealed substantial label–composition discordance, including negligible amounts of turmeric‐derived constituents in a *Curcuma longa* formulation and multiple undeclared synthetic drugs in a weight‐loss product. The integration of structured clinical assessment with product verification refined causal attribution and, in selected cases, supported the reclassification of suspected herbal hepatotoxicity as probable adulteration‐related drug‐induced liver injury.

**Conclusion:**

Product authentication may substantially improve the diagnostic accuracy of suspected HILI and reduce the etiological uncertainty. Incorporating the verification of consumed supplements into hepatology evaluation may help distinguish true herbal hepatotoxicity from adulteration‐related liver injury, thereby improving patient safety and pharmacovigilance.

## 1. Introduction

The global use of herbal and dietary supplements (HDS) has increased substantially in recent decades, accompanied by a growing number of reports of herb‐induced liver injury (HILI) [1, 2]. Although frequently perceived as safe or “natural,” these products represent an increasingly recognized cause of liver injury encountered in clinical hepatology practice [3]. Variability in composition, limited regulatory oversight, and potential presence of undeclared pharmacologically active substances complicate diagnostic evaluations and may obscure causal attributions [4, 5].

Establishing causality in suspected HILI remains particularly challenging [6]. The updated Roussel Uclaf Causality Assessment Method (RUCAM, 2016) provides a structured and validated framework for assessing drug‐ and supplement‐related liver injuries [7, 8]. However, accurate classification is often limited by incomplete exposure data, concomitant medications, metabolic comorbidities, and uncertainty regarding the authenticity and composition of consumed products [6, 8]. Discrepancies between the labelled and actual product contents may lead to the misclassification of liver injury as herbal toxicity rather than adulteration‐related drug‐induced liver injury [4, 9].

Increasingly, analytical verification of implicated supplements has been proposed as a complementary strategy for clinical assessment, particularly when diagnostic uncertainty persists [10, 11]. However, systematic integration of clinical causality assessment with direct analysis of consumed products in real‐world HILI cases remains limited [10, 12, 13].

In this context, we describe a clinically characterized case series of patients with suspected HILI in whom a structured causality assessment was complemented by analytical authentication of the consumed supplements. This approach aimed to evaluate whether verification of product composition could refine causal attribution and improve the diagnostic interpretation of suspected supplement‐related liver injury.

## 2. Case Presentation and Methods

This observational case series describes patients with suspected herb‐ and dietary supplement–induced liver injury evaluated using a structured clinical causality assessment, complemented by an analytical evaluation of the consumed products. Case reporting followed CARE recommendations to ensure transparency and completeness [14].

Five patients with suspected HILI were evaluated at the Professor Edgard Santos University Hospital (HUPES), Federal University of Bahia (UFBA), Salvador, Brazil, between December 2023 and September 2024. The patients were referred by the gastroenterology service and assessed during hospitalization and outpatient follow‐ups. This study was approved by the Institutional Research Ethics Committee (CAAE: 79080224.7.0000.0049).

Patients were included if they presented with clinically significant liver injury following exposure to herbal products or dietary supplements, defined as alanine aminotransferase elevation ≥ 5× the upper limit of normal, alkaline phosphatase elevation ≥ 2× the upper limit of normal, or compatible mixed injury patterns [3, 8]. Alternative etiologies were excluded according to standard hepatology evaluation protocols, including viral hepatitis serology, autoimmune markers, and imaging studies.

Clinical follow‐up included serial monitoring of liver biochemistry, symptom assessment, documentation of comorbidities, and a review of concomitant medications. Written informed consent was obtained from all participants and suspected products were obtained directly from patients or relatives, for subsequent analytical verification (Table 1).

**TABLE 1:** Herbal products and dietary supplements included in the study.

Case	Sample type	Labelled composition
CQLM	Raw herbal (polyherbal tea)	Cow’s foot (*Bauhinia* spp., leaves), hibiscus (*Hibiscus sabdariffa*, calyces), cinnamon (*Cinnamomum* spp., bark)
EFJ	Herbal and dietary supplements (HDS)–oil extract	Turmeric oil (*Curcuma longa*, rhizome) with collagen
TSG	Raw herbal drugs (herbal teas)	Dandelion (*Taraxacum officinale*, leaves/aerial parts), horsetail (*Equisetum arvense*, sterile aerial stems), rosemary (*Rosmarinus officinalis*, leaves), hibiscus (*Hibiscus sabdariffa*, calyces)
GSL	Weight‐loss herbal and dietary supplements (HDS)–capsules	Konjac glucomannan (*Amorphophallus konjac*, tuber extract), cinnamon bark extract (*Cinnamomum* spp.), and declared nutraceutical/mineral constituents (HMB, magnesium, taurine, zinc, phosphatidylserine, potassium iodide, tryptophan, caffeine, chromium picolinate)
RGL	Raw herbal product (herbal tea)	Moringa (*Moringa oleifera*, leaves)

**FIGURE 1:**
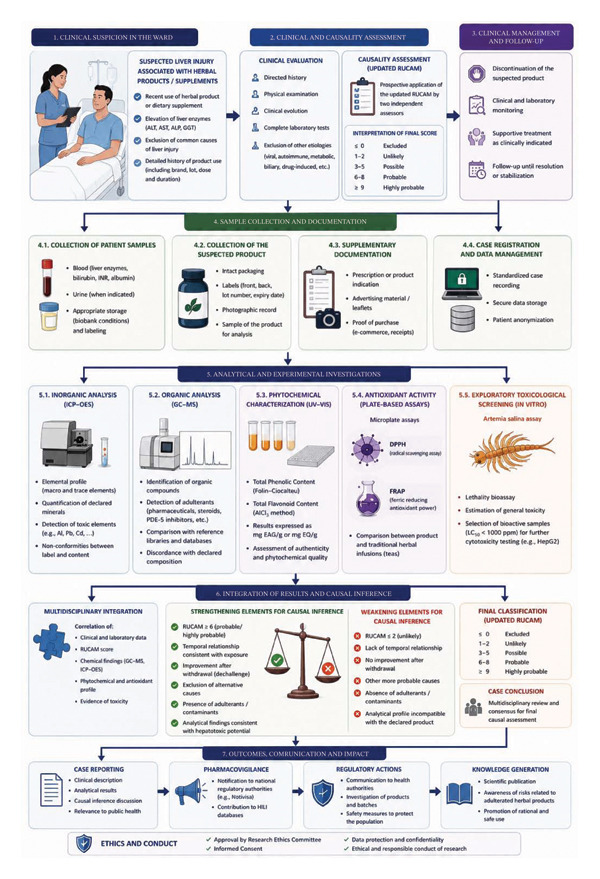
Translational workflow integrating clinical evaluation, updated RUCAM causality assessment, chemical authentication, and exploratory bioactivity analyses in suspected herb‐related liver injury.

The updated RUCAM was applied prospectively during clinical evaluation and follow‐up using contemporaneously collected clinical, biochemical, and imaging data obtained during hospitalization and outpatient assessments. Variables included latency, biochemical evolution after product withdrawal, risk factors, concomitant medications, and exclusion of competing etiologies. The overall clinical, analytical, and translational workflow applied in the present study is summarized in Figure 1.

Chemical authentication and exploratory bioactivity assessment of the selected herbal and commercial formulations were performed to provide complementary support for clinical causality assessment. Chemical composition and toxicity screening assays were conducted under standardized laboratory conditions [4, 10, 11]. The detailed analytical methodologies are provided in the Supporting Methods section.

## 3. Results

### 3.1. Clinical and Demographic Characteristics

This case series included five patients evaluated at a tertiary hepatology referral center who required inpatient assessment followed by outpatient clinical follow‐up. The patients ranged in age from 39 to 64 years, with a predominance of females (4/5). Overweight or obesity was present in four patients. Relevant comorbidities, including metabolic syndrome, type 2 diabetes mellitus, hypertension, dyslipidemia, and obesity, are recognized as potential modifiers of susceptibility to liver injury.

Pre‐existing liver disease or prior hepatic events were documented in three patients, representing possible cofactors influencing clinical presentation and causality interpretation. Alcohol consumption was absent or occasional in most cases. Concomitant medication exposure varied and included the use of antihypertensive agents, oral hypoglycemic drugs, and intermittent nonsteroidal anti‐inflammatory drugs.

The individual clinical summaries below emphasize exposure history, clinical presentation, exclusion of competing etiologies, and clinical outcomes. The detailed demographic and clinical characteristics are presented in Table 2.

**TABLE 2:** Clinical and demographic characteristics of patients with suspected herb‐induced liver injury (HILI).

Case	Age	Sex	BMI kg/m^2^	Comorbidity	Previous liver history	Alcohol use	Concomitant medications
CQLM	52	Female	34.9	Class I obesity; metabolic syndrome	Autoimmune hepatitis	No	Antihypertensive agents
EFJ	64	Female	29.4	Hypertension; Type 2 diabetes mellitus; congenital mitral valve disease; umbilical hernia	Chronic liver parenchymal disease	No	Antihypertensive; NSAIDs
TSG	39	Female	—	None documented	Previous viral hepatitis; prior DILI episode (anabolic steroids)	No	None
GSL	39	Female	34.6	Class I obesity	No relevant prior liver disease	Occasional social use	None
RGL	58	Male	29.7	Type 2 diabetes mellitus; Class I obesity; dyslipidemia; metabolic syndrome	MASLD	Weekend consumption	Oral hypoglycemic agents

*Note:* NSAIDs, nonsteroidal anti‐inflammatory drugs; MASLD, metabolic dysfunction–associated steatotic liver disease. Alcohol consumption was classified according to the patients’ self‐reports.

Abbreviations: BMI, body mass index; DILI, drug‐induced liver injury.

### 3.2. Individual Case Descriptions

#### 3.2.1. Case 1 (CQLM)

A 52‐year‐old woman with metabolic syndrome, obesity, hypertension, and autoimmune hepatitis (AIH) was in clinical remission before herbal exposure. The patient presented with progressive cholestatic symptoms including pruritus, jaundice, dark urine, fatigue, dyspepsia, weight loss (∼15 kg), and episodic mental confusion. She reported the consumption of herbal infusions containing *Hibiscus sabdariffa*, *Bauhinia* spp., and *Cinnamomum* spp. approximately 45 days before symptom onset.

Laboratory evaluation revealed cholestatic liver injury with elevated aminotransferases, conjugated hyperbilirubinemia, and a prolonged INR. Imaging studies have excluded biliary obstructions. Liver biopsy revealed advanced AIH with cirrhosis (A3F4), intense inflammatory activity, and submassive necrosis. A partial clinical improvement was observed after corticosteroid therapy. The patient remains under hepatology follow‐up.

#### 3.2.2. Case 2 (EFJ)

A 64‐year‐old woman with hypertension, type 2 diabetes mellitus, and congenital mitral valve disease developed abdominal pain, dyspeptic symptoms, pruritus, choluria, and anorexia shortly after initiation of an unregulated turmeric oil–based supplement combined with collagen. She also reported frequent nonsteroidal anti‐inflammatory (NSAID) use, which was considered a potential competing exposure during causality assessment.

Laboratory tests showed marked hepatocellular injury (AST 2420 U/L; ALT 2460 U/L) with superimposed cholestatic features. Imaging findings suggested underlying chronic liver disease with ascites and collateral circulation. Viral hepatitis screening and autoimmune marker test results were negative or inconclusive. Corticosteroid therapy resulted in partial biochemical improvement, although cholestasis persisted during follow‐up.

#### 3.2.3. Case 3 (TSG)

A 39‐year‐old woman with obesity and dyslipidemia underwent hepatologic evaluation after clinically relevant liver enzyme abnormalities were identified during a medical assessment because of nonspecific systemic complaints and ongoing use of herbal products. She reported regular consumption of mixed herbal infusions including *Hibiscus sabdariffa* and prior exposure to nonprescribed anabolic steroids.

Laboratory findings predominantly indicated hepatocellular injury. Patients with viral, autoimmune, or structural causes were excluded from this study. The discontinuation of herbal products was followed by progressive normalization of liver enzyme levels without specific pharmacological intervention, supporting a possible association with herbal exposure.

#### 3.2.4. Case 4 (GSL)

A previously healthy 39‐year‐old woman presented with acute neurological symptoms (vomiting, transient loss of consciousness, muscle jerks, and dysarthria), associated with severe liver enzyme elevation and rhabdomyolysis (marked creatine kinase elevation and myoglobinuria).

Extensive etiological investigations have excluded viral hepatitis, systemic infections, and structural hepatobiliary disease. The directed history indicated the recent use of a commercially available weight‐loss dietary supplement for 5 days. Supportive care led to clinical recovery and normalization of laboratory parameters after discontinuation.

#### 3.2.5. Case 5 (RGL)

A 58‐year‐old man with type 2 diabetes, metabolic syndrome, dyslipidemia, and a known metabolic‐associated fatty liver disease presented with chronic mild liver enzyme abnormalities during outpatient monitoring. He reported regular consumption of *Moringa oleifera* tea for glycemic control.

Despite continued biochemical abnormalities, no significant worsening occurred during exposure. The biochemical profile remained consistent with the underlying MASLD without a clear temporal association with herbal intake. The discontinuation of herbal tea did not significantly alter liver enzyme levels.

### 3.3. Causality Assessment Using the Updated RUCAM

The updated RUCAM‐based causality assessment indicated that four of the five cases fulfilled the criteria for at least possible HILI, whereas one case was classified as unlikely (Table 3).

**TABLE 3:** Updated RUCAM‐based causality assessment, liver injury pattern, and suspected products in cases of herb‐ and dietary supplement–induced liver injury (HILI).

Case	Suspected product/use	*R* ratio	Liver injury pattern	Updated RUCAM score	Causality level	Proposed mechanism
CQLM	Polyherbal‐tea preparation	4.09	Mixed	4	Possible	Idiosyncratic (metabolic)
EFJ	Turmeric oil with collagen (oral use, diluted in water)	14.04	Hepatocellular	5	Possible	Idiosyncratic (metabolic)
TSG	Herbal mixture and isolated herbal teas	13.30	Hepatocellular	5	Possible	Idiosyncratic (metabolic)
GSL	Weight‐loss HDS capsules	87.76	Hepatocellular	9	Highly probable	Intrinsic (adulteration‐related toxicity)
RGL	Single‐herb tea preparation	1.64	Cholestatic	−3	Unlikely	Indeterminate

*Note: R* ratio, ALT/ULN divided by ALP/ULN, used to classify liver injury patterns as hepatocellular (*R* ≥ 5), cholestatic (*R* ≤ 2), or mixed (*R* > 2 and < 5). Causality grading was prospectively performed according to updated RUCAM criteria (excluded, unlikely, possible, probable, and highly probable).

Abbreviations: HDS, herbal and dietary supplements; HILI, herb‐induced liver injury; RUCAM, Roussel Uclaf Causality Assessment Method.

The liver injury pattern, defined by the *R* ratio (ALT/ULN ÷ ALP/ULN), was predominantly hepatocellular and observed in three patients (EFJ, TSG, and GSL), whereas a mixed pattern was identified in CQLM and a cholestatic pattern in RGL.

The GSL case yielded the highest causality score (updated RUCAM = 9, highly probable), which significantly exceeded the scores observed in the remaining cases.

Based on clinical evolution and exposure characteristics, most cases were clinically compatible with idiosyncratic liver injury, whereas the GSL case showed features suggestive of intrinsic‐like toxicity associated with suspected exposure to undeclared compounds. The RGL case was classified as unlikely according to the updated RUCAM, mainly because of the presence of competing metabolic liver disease.

Overall, the heterogeneity observed across the cases, including differences in liver injury patterns, causality scores, and exposure profiles, reinforces the multifactorial nature of suspected HILI. The integration of structured clinical assessment with complementary analytical investigations has contributed to a more comprehensive interpretation of HILI/DILI.

## 4. Discussion

### 4.1. Clinical Features and Causality Assessment in Suspected HILI Cases

This case series integrates clinical assessment with complementary analytical investigations to improve causality evaluation of suspected HILI [4, 6, 7]. A recurrent clinical profile has emerged, predominantly involving adult women who are overweight and have metabolic syndrome and related comorbidities (CQLM, EFJ, and TSG) [1, 12, 13].

Such conditions have been described as susceptibility factors for xenobiotic‐induced liver injury, because metabolic dysfunction may alter hepatic drug disposition, inflammatory signaling, and adaptive stress responses, favoring idiosyncratic injury patterns [1, 3, 8].

Cases CQLM and EFJ highlight the diagnostic complexity of distinguishing HILI from underlying chronic liver diseases, particularly AIH [3, 8, 15].

In CQLM, prolonged herbal exposure preceded severe cholestatic hepatitis and subsequent histological confirmation of advanced AIH, suggesting a possible triggering effect of herbal products on the latent autoimmune processes. This aligns with the concept of drug‐induced autoimmune‐like hepatitis (DI‐AILH), in which xenobiotics act as immune triggers without necessarily requiring long‐term immunosuppression or causing recurrence after withdrawal [8, 15].

The systematic use of the updated RUCAM enabled structured causality stratification and reduced subjective interpretations across the five cases (see Table S1) [7]. The patient with GSL achieved a high score, strongly supporting a causal link between the adulterated weight‐loss product (“Gym Power–Gold”) and hepatic injury, consistent with reports of undeclared pharmacologically active substances in similar products [4, 9, 11].

Detailed chromatographic profiles and fragmentation spectra of the weight‐loss product revealed the presence of multiple undeclared synthetic pharmaceuticals, including centrally acting agents, consistent with controlled or prescription‐only use, clearly demonstrating intentional product adulteration. This finding provides a mechanistic basis for the high causality score observed with the updated RUCAM, as the concomitant exposure to multiple pharmacologically active, undeclared compounds, particularly those with known hepatotoxic potential, substantially increases the likelihood of liver injury and strengthens causal attribution [4, 9–11, 16].

In contrast, the RGL case illustrates the limitations of attribution in the presence of MAFLD, alcohol use, and chronic hepatotoxic medications, resulting in an “unlikely” classification. Case TSG represented an intermediate scenario (“possible”), complicated by metabolic comorbidities and prior anabolic steroid exposure [17].

Collectively, these findings emphasize that HILI arises from the interplay between product exposure, host metabolic profile, and pre‐existing liver conditions rather than intrinsic toxicity alone. Notably, higher causality scores were associated with products lacking regulatory oversight or presenting compositional irregularities, as was observed in the GSL case [1, 8, 18].

Despite reliance on case series and retrospective analyses, the broader literature confirms that herb‐ and supplement‐associated liver injury is clinically relevant and potentially severe [12, 13].

Reports involving large case compilations have documented significant outcomes including hospitalization, liver transplantation, and mortality, even for products that are widely perceived as safe [12, 13]. These observations align with the findings of the present study, in which the irregular chemical composition of the consumed supplements and patient metabolic vulnerability appeared to converge with the clinical presentation of liver injury.

### 4.2. Comparison With Published HILI Cases Assessed by RUCAM

Previous reports of herb‐ and dietary supplement–induced liver injury assessed using either the traditional or updated RUCAM have described heterogeneous clinical presentations ranging from mild hepatocellular injury to acute liver failure requiring transplantation (Table 4) [3, 8].

**TABLE 4:** Summary of selected representative HILI reports evaluated using traditional or updated RUCAM approaches.

Study	Product/exposure	Injury pattern	RUCAM version	Causality	Key findings
Navarro et al., 2019	Multiple HDS	Hepatocellular	Traditional	Probable–highly probable	Frequent mislabelling/adulteration
Bessone et al., 2022	Latin American HILI registry	Mixed	Updated/traditional	Variable	Severe outcomes reported
de Assis et al., 2022	Pooled HILI case reports	Predominantly hepatocellular	Traditional	Possible–highly probable	High heterogeneity
Sacundino et al., 2022	*Moringa oleifera*	Hepatocellular	Updated	Probable	Positive rechallenge
Present study	Herbal teas and adulterated HDS	Predominantly hepatocellular	Updated	Possible–highly probable	High‐confidence causal attribution supported by integrated clinical, chemical, and toxicological evidence

*Note:* Representative studies were selected to illustrate the application of a structured causality assessment in published HILI reports involving herbal products and dietary supplements.

Abbreviations: HDS, herbal and dietary supplements; HILI, herb‐induced liver injury; RUCAM, Roussel Uclaf Causality Assessment Method.

Similar to the present series, several published cases involved products marketed for weight loss, metabolic control, or wellness purposes, which are frequently affected by compositional variability or adulteration [9, 11]. Structured causality assessment using the updated RUCAM has become increasingly important in HILI investigations because it improves diagnostic standardization and reduces subjective attribution.

### 4.3. Chemical Authenticity as a Determinant of HILI Risk

Undeclared hepatotoxic compounds in products marketed as “natural” supplements are major determinants of HILI and act as primary causal agents or aggravating factors [4, 9, 11]. In this context, chemical characterization is critical for interpreting clinical findings. Notably, only the commercially manufactured products included in this case series (EFJ‐1 and GSL) exhibited substantial discrepancies between labelled and detected compositions, including the absence of expected botanical constituents and the presence of multiple undeclared synthetic drugs in the GSL formulation, as summarized in Table 5.

**TABLE 5:** Label‐declared composition versus analytical findings (GC–MS and ICP–OES) in two manufactured products associated with suspected liver injury (EFJ‐1 and GSL cases).

Sample	Category	Label‐declared composition	Analytical findings (GC–MS/ICP–OES)	Interpretation
Turmeric oil with collagen–EFJ case	Botanic constitutions	Curcumin	α‐Turmerone and β‐turmerone trace ∼0.61%; curlone ∼0.52%; curcuminoids not detected by GC–MS	Lipophilic chemical profile characterized with only trace turmeric sesquiterpenes; suggestive of absence of botanical authenticity
	Protein component	Collagen	No proteinaceous markers detected (GC–MS not suitable for protein confirmation)	Absence of confirmatory analytical evidence for collagen under applied methodology

Weight‐loss HDS–GSL case	Botanicals and nutraceuticals	Konjac (*Amorphophallus konjac*), cinnamon (*Cinnamomum verum*), HMB (β‐hydroxy‐β‐methylbutyrate), taurine, phosphatidylserine, tryptophan, caffeine	Not detected	Findings consistent with mislabelling and the absence of declared botanical/nutraceutical constituents

	Synthetic drugs	Not declared	Sibutramine (RT 25.6 min; 75.81%; ∼247 mg); N‐acetyl fluoxetine (RT 33.4 min; 3.89%; 30.9 mg); fluoxetine (RT 26.1 min; 2.64%; ∼21 mg); bisacodyl (RT 42.5 min; 4.16%; ∼14 mg); diazepam (RT 36.5 min; 2.01%; ∼6.5 mg); bupropion (RT 20.1 min; 0.80%; ∼2.6 mg).	The presence of multiple undeclared pharmacologically active substances, consistent with product adulteration and suggesting potential hepatotoxic risk

	Minerals	Magnesium (Mg), zinc (Zn), potassium (K), chromium picolinate (Cr)	Zn: 16–17 ppm; K: 2.6%–2.9% w/w; Mg: ∼0.17% w/w; Al: ∼300 ppm (undeclared); Pb, Cr < LOD	Partial agreement with the label; the detection of undeclared aluminum and the absence of chromium

*Note:* The limitations of GC–MS include the poor detection of high‐molecular‐weight proteins, such as collagen.

Abbreviation: LOD, limit of detection.

Product marketed as “turmeric oil with collagen” was found to consist predominantly of fatty acid esters and triacylglycerols, with only trace amounts of turmeric‐derived constituents. Although GC–MS is not suitable for detecting high‐molecular‐weight proteins such as collagen, the absence of characteristic low‐molecular‐weight markers associated with collagen‐derived products, together with the lack of curcuminoids (e.g., curcumin) and the minimal presence of turmeric chemomarkers, indicates a clear inconsistency with the labelled composition [4]. These findings support the lack of chemical authenticity of EFJ1.

The total ion chromatogram of the EFJ‐1 sample (EFJ case) is shown in Figure S1, and the spectral identification of turmerone and curlone is detailed in Figures S2 and S3, respectively.

**FIGURE 2:**
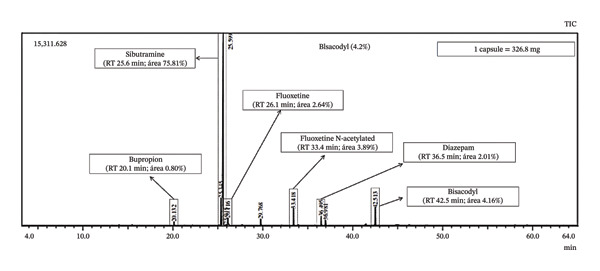
GC–MS chromatogram of the analyzed product indicating major undeclared synthetic pharmaceutical compounds (“Gym Power” capsules–GSL case).

In contrast, GC–MS analysis of the GSL sample revealed a complex chemical profile dominated by undeclared pharmacologically active substances (Figure S4). The total ion chromatograms and peak distributions are shown in Figure 2. The mass spectral confirmation of sibutramine is presented in Figure S5. These compounds were identified as major constituents, rather than trace contaminants, supporting the likelihood of intentional adulteration.

The analytical findings demonstrated the presence of multiple synthetic drugs, including fluoxetine, bupropion, diazepam, bisacodyl, and sibutramine, consistent with the extensive compositional irregularities observed in the weight‐loss product. The combined pharmacological profile supports a synergistic mechanism involving dopaminergic stimulation, cytochrome P450 (CYP)‐mediated interactions, oxidative stress, and mitochondrial dysfunction [13, 19].

In addition, elemental analysis revealed elevated undeclared aluminum and chromium concentrations below the limit of detection despite label claims (Table S2), further reinforcing concerns regarding product authenticity and safety [9, 11]. From a clinical perspective, the GSL case was characterized by acute hepatocellular injury with marked aminotransferase elevation, rapid biochemical improvement after product withdrawal, and a highly updated RUCAM score, collectively supporting adulteration‐related drug‐induced liver injury.

Notably, one of the cases included in the present translational case series was previously reported individually as the Gym Power case report published in Toxicology Reports, which integrated clinical, chemical, and toxicological evidence to support a highly probable diagnosis of adulteration‐related liver injury according to the updated RUCAM (2016) [20]. That report also demonstrated the presence of multiple undeclared synthetic compounds in the product, reinforcing the hypothesis that the observed hepatotoxicity was associated with complex pharmacological interactions rather than intrinsic botanical toxicity. In the present study, this case is re‐evaluated within a broader translational framework including comparative phytochemical authentication and causality assessment across multiple herbal products.

In contrast, the RGL sample (*Moringa oleifera*) exhibited phytochemical characteristics compatible with botanical authenticity, low toxicity in the exploratory assays, and an “unlikely” classification according to the updated RUCAM, highlighting the relevance of complementary analytical characterization in causality assessment. These findings further suggest that in some published reports, the absence of phytochemical verification may limit the distinction between intrinsic herbal toxicity, compositional variability, and potential adulteration‐related effects [21, 22].

Therefore, chemical characterization provides critical support for clinical causality assessment and helps to distinguish true herbal hepatotoxicity from exposure to undeclared pharmaceuticals, with direct implications for patient safety, pharmacovigilance, and regulatory oversight [4, 7, 8].

In addition to the identification of exogenous adulterants, complementary phytochemical analyses are essential for further evaluating the composition and bioactivity of these products.

To further contextualize the analytical findings and their potential clinical relevance, complementary phytochemical characterization and antioxidant activity assays were performed.

### 4.4. Phytochemical Characterization, Antioxidant Activity, and Clinical Implications

Phytochemical screening revealed substantial variability in phenolic and flavonoid content among the investigated products, reflecting differences in botanical authenticity, processing, and formulation standardization [4, 5, 9]. Quantitative spectrophotometric data are shown in Figures S6–S9, including calibration curves (Figures S6 and S8) and total phenolic and flavonoid content (Figures S7 and S9).

Commercial formulations (EFJ and GSL) exhibited the lowest phenolic and flavonoid levels, consistent with chemical mischaracterization, whereas traditional herbal preparations (CQLM, TSG, and RGL) showed phytochemical profiles compatible with plant‐derived matrices.

Antioxidant activity assessed by 2,2‐diphenyl‐1‐picrylhydrazyl (DPPH) and ferric reducing antioxidant power (FRAP) assays followed similar variability patterns, but did not correlate with clinical safety, as these assays primarily reflect chemical reactivity rather than biological availability or hepatotoxic potential [19, 23]. Antioxidant activity was evaluated using complementary assays and is presented in Figures S10–S12.

Specifically, Figure S10 shows the DPPH radical scavenging activity of the herbal materials and commercial formulations (EFJ‐1, turmeric oil with collagen; GSL, weight‐loss product), expressed as a percentage of inhibition or antioxidant capacity.

Figure S11 shows the calibration curve used for the FRAP assay (ferrous sulfate; *y* = 0.0002*x* + 0.086; *R* ^2^ = 0.9984), confirming the linearity and analytical reliability of the method. Figure S12 shows the FRAP of commercial HDS (EFJ‐1 and GSL) and herbal hydroethanolic extracts (10% w/v) based on Fe^3+^–TPTZ reduction. Overall, the results indicated reduced antioxidant activity in HDS compared to herbal materials and traditional preparations. Previous studies have similarly demonstrated that products marketed as “natural” may display wide compositional variability independent of safety outcomes [13, 19].

From a clinical perspective, these findings reinforce that the phytochemical composition alone cannot predict the risk of hepatotoxicity. In the present cases, liver injury was more plausibly associated with marked chemical inconsistency and, in some instances, the presence of undeclared pharmacologically active synthetic substances identified by GC–MS analysis [3, 4, 9].

These observations support a multifactorial mechanism involving product adulteration, compositional variability, and host susceptibility, rather than the expected effects of labelled herbal ingredients alone [23, 24]. Accordingly, phytochemical and antioxidant measurements should be interpreted as supportive contextual data, rather than as determinants of causality [19].

Experimental bioactivity assays provide complementary translational information regarding the biological behavior of products consumed by patients [23, 25, 26]. Importantly, when combined with analytical and clinical findings, these approaches contribute to a more comprehensive assessment of the potential risks associated with chemically inconsistent or adulterated formulations.

### 4.5. In Vitro Toxicity as a Translational Tool

Exploratory in vitro toxicity screening provided a supportive biological context for the variability among the investigated products. The assay revealed marked variability among the authentic herbs, mixtures, and industrial formulations.

Notably, both industrial products (EFJ‐1 and GSL) demonstrated higher cytotoxicity profiles, consistent with their chemical complexity and previously identified compositional irregularities, whereas most isolated herbal preparations exhibited low acute toxicity. Notably, *Hibiscus sabdariffa* flowers exhibited one of the highest toxicity profiles in the *Artemia salina* bioassay, with an LC_50_ value below 100 ppm. This botanical ingredient was consumed by two patients included in the present series (CQLM and TSG), both as an isolated herbal preparation and in combination with other medicinal plants prepared as traditional infusions (teas). The elevated toxicity observed under both exposure conditions suggests that Hibiscus‐containing preparations may contribute substantially to the overall biological activity of these formulations. Given the widespread consumption of *Hibiscus sabdariffa* for weight‐loss and wellness purposes, these findings reinforce the need for further toxicological and clinical investigations to better characterize its safety profile in susceptible individuals. Comparative toxicity results are presented in Figure S13 and Table S3. Herbal mixtures tended to display greater toxicity than individual plant preparations, suggesting possible additive or synergistic biological effects that may better reflect real‐world exposure scenarios [23, 26].

However, given the nonorgan‐specific nature of this model, these findings should be interpreted cautiously and not as direct indicators of hepatotoxicity. Rather, the experimental results provide a complementary biological perspective for clinical observations by highlighting the variability in bioactivity of products consumed by patients [19, 26].

When interpreted alongside structured clinical causality assessments and chemical authentication, such exploratory assays may contribute to a broader translational framework for investigating suspected herb‐ and dietary supplement–associated liver injuries.

### 4.6. Implications for Pharmacovigilance of Irregular Products Marketed as Herbal and Dietary Supplements

The present case series illustrates that integrating structured clinical causality assessment with chemical characterization and exploratory bioactivity evaluation may provide additional context for interpreting suspected cases of herb‐ and dietary supplement–induced liver injury [3, 7, 8]. Such complementary approaches may be particularly valuable in clinical scenarios involving multiple confounding factors and uncertain product composition.

These findings highlight the importance of collaboration between clinicians, analytical laboratories, and pharmacy services when evaluating patients exposed to nonregulated or poorly standardized supplements, as “natural” labelling or antioxidant claims do not reliably predict safety [4, 9].

Chemically complex or adulterated products may contribute to clinically significant liver injury, independent of their declared botanical identity, as supported by discrepancies between labelled and observed compositions [11]. The investigated products included two commercial formulations (EFJ‐1 and GSL), with the labeling and packaging characteristics documented in Figure S14.

From a pharmacovigilance perspective, the documentation of product exposure, verification of composition when feasible, and structured causality assessments may improve diagnostic confidence and support safety reporting [8, 13, 25]. Pharmacists and clinical teams play relevant roles in exposure documentation, coordination of analytical investigations, and communication regarding suspected adverse events.

Despite the inherent limitations of small case series and exploratory experimental models, the integration of clinical observations with analytical data provided coherent convergent evidence for etiological interpretation of the present cases. This integrative approach also highlights the importance of systematic documentation and cross‐disciplinary collaboration to strengthen causality assessments in complex exposure scenarios. Collectively, these observations reinforce the value of multidisciplinary evaluation in improving the recognition and reporting of HDS‐associated liver injury in real‐world clinical practice.

## 5. Conclusion

This case series describes five patients with suspected herb‐ and dietary supplement–induced liver injury evaluated at a tertiary hepatology referral center, highlighting the diagnostic complexity associated with heterogeneous clinical backgrounds, metabolic comorbidities, and pre‐existing liver disease. Systematic application of the updated RUCAM enabled a structured causality assessment with classifications ranging from possible to highly probable across cases.

The integration of hepatology evaluation with targeted analytical characterization provided important contextual information for the interpretation of selected cases. Chemical analyses identified substantial discrepancies between the labelled and detected compositions, including botanical mischaracterization and undeclared synthetic compounds, particularly among commercially manufactured products with higher causality scores.

These findings suggest that complementary analytical investigations may assist in identifying suspected HILI when product authenticity or composition is uncertain. Accurate etiological attribution remains essential in hepatology practice because it directly influences exposure withdrawal, clinical management, and pharmacovigilance reporting. Multidisciplinary approaches integrating hepatology, pharmacovigilance, and analytical authentication may strengthen causality assessment and improve the recognition of chemically mischaracterized or adulterated products in real‐world HILI investigations.

## Funding

This work was supported by the Coordination for the Improvement of Higher Education Personnel (CAPES), Brazil (finance code 001; grant number 88887.920343/2023‐00).

## Disclosure

The funders had no role in the study design, data collection and analysis, decision to publish, or manuscript preparation.

## Consent

Written informed consent was obtained from the patients for the publication of this case series and accompanying clinical data. The consent forms were securely archived by the authors and are available for editorial review upon request.

## Conflicts of Interest

The authors declare no conflicts of interest.

## Supporting Information

Additional supporting information can be found online in the Supporting Information section.

## Supporting information


**Supporting Information** *Supporting Table and Figure Legends*. Table S1: Individual scoring components of the updated RUCAM applied to cases of suspected HILI. Table S2: Elemental profiling of the weight‐loss product (GSL sample) by ICP–OES. Table S3: Toxicity screening of aqueous extracts of herbal materials and industrialized products marketed as herbal dietary supplements (HDS), including preparations traditionally consumed as infusions (teas), from samples associated with HILI. Figure S1: GC–MS chromatogram of the commercial formulation “Curcumine” (EFJ‐1 sample). Figure S2: Chromatographic identification by GC‐MS of the chemomarker tumerone (EFJ‐1 sample). Figure S3: Chromatographic identification by GC‐MS of the chemomarker curlone (EFJ‐1 sample). Figure S4: GC–MS chromatogram of the weight‐loss product (GSL sample). Figure S5: Chromatographic identification by GC–MS of the major adulterant (sibutramine) in the weight‐loss product (GSL sample). Figure S6: Gallic acid calibration curve obtained by UV–Vis spectrophotometry (*y* = 0.0044*x* + 0.0863; *R* ^2^ = 0.9975) used for quantification of total phenolic content (gallic acid equivalents, GAE). Figure S7: Total phenolic content of herbal materials collected from patients and prepared as hydroethanolic extracts (10% w/v). Figure S8: Quercetin calibration curve obtained by UV–Vis spectrophotometry (*y* = 0.0032*x* + 0.0012; *R* ^2^ = 0.9982) used for quantification of total flavonoid content (quercetin equivalents, QE). Figure S9: Total flavonoid content of herbal materials collected from patients and prepared as hydroethanolic extracts (10% w/v). Figure S10: Antioxidant activity of herbal materials and industrialized products marketed as HDS (EFJ‐1, turmeric oil with collagen; GSL, weight‐loss product), determined by the DPPH radical scavenging assay. Figure S11: Ferric reducing antioxidant power (FRAP) of herbal materials, traditional herbal infusions (teas), and commercially manufactured products marketed as herbal formulations. Antioxidant capacity was determined using the FRAP assay and quantified based on a ferrous sulfate calibration curve (*y* = 0.0002*x* + 0.086; *R* ^2^ = 0.9984). Figure S12: Ferric reducing antioxidant power (FRAP) of herbal materials and commercially manufactured products marketed as herbal formulations (EFJ‐1 and GSL). Samples were prepared as hydroethanolic extracts at 10% (w/v) prior to analysis. Figure S13: Comparative LC_50_ values (ppm) of aqueous herbal extracts and industrialized products marketed as herbal formulations (EFJ‐1, turmeric oil with collagen; GSL, weight‐loss product), evaluated at 5% (w/v) using the *Artemia salina* lethality bioassay (*n* = 120 nauplii). Figure S14: Photographic documentation of two commercially manufactured products marketed as HDS and suspected of hepatotoxicity.

## Data Availability

The data supporting the findings of this study are available from the corresponding author upon reasonable request.
