# Supplementary material for: Liver Injury Associated With Irregular Herbal Products: A Translational Case Series With Chemical Authentication and Causality Assessment Using the Updated RUCAM
Source: Case Reports Hepatol. 2026 Jul 17;2026:5905774. doi: 10.1155/crhe/5905774 (PMC13377616; doi:10.1155/crhe/5905774)
Supplement: Supplementary file 1 — Supporting Information Supporting Table and Figure Legends. Table S1: Individual scoring components of the updated RUCAM applied to cases of suspected HILI. Table S2: Elemental profiling of the weight‐loss product (GSL sample) by ICP–OES. Table S3: Toxicity screening of aqueous extracts of herbal materials and industrialized products marketed as herbal dietary supplements (HDS), including preparations traditionally consumed as infusions (teas), from samples associated with HILI. Figure S1: GC–MS chromatogram of the commercial formulation “Curcumine” (EFJ‐1 sample). Figure S2: Chromatographic identification by GC‐MS of the chemomarker tumerone (EFJ‐1 sample). Figure S3: Chromatographic identification by GC‐MS of the chemomarker curlone (EFJ‐1 sample). Figure S4: GC–MS chromatogram of the weight‐loss product (GSL sample). Figure S5: Chromatographic identification by GC–MS of the major adulterant (sibutramine) in the weight‐loss product (GSL sample). Figure S6: Gallic acid calibration curve obtained by UV–Vis spectrophotometry (y = 0.0044x + 0.0863; R 2 = 0.9975) used for quantification of total phenolic content (gallic acid equivalents, GAE). Figure S7: Total phenolic content of herbal materials collected from patients and prepared as hydroethanolic extracts (10% w/v). Figure S8: Quercetin calibration curve obtained by UV–Vis spectrophotometry (y = 0.0032x + 0.0012; R 2 = 0.9982) used for quantification of total flavonoid content (quercetin equivalents, QE). Figure S9: Total flavonoid content of herbal materials collected from patients and prepared as hydroethanolic extracts (10% w/v). Figure S10: Antioxidant activity of herbal materials and industrialized products marketed as HDS (EFJ‐1, turmeric oil with collagen; GSL, weight‐loss product), determined by the DPPH radical scavenging assay. Figure S11: Ferric reducing antioxidant power (FRAP) of herbal materials, traditional herbal infusions (teas), and commercially manufactured products marketed as herbal formulation [file CRHE-2026-5905774-s001.docx]

**Supplementary Material**

**Tables**

**Table S1.** Individual scoring components of the updated RUCAM (Roussel Uclaf Causality Assessment Method) applied to cases of suspected herb-induced liver injury (HILI). Each domain was scored according to established criteria, and total scores were used to determine causality likelihood. Causality grading: ≤0 (excluded), 1–2 (unlikely), 3–5 (possible), 6–8 (probable), ≥9 (highly probable).

| **Case** | **Time to onset** | **Course after withdrawal** | **Risk factors** | **Concomitant drugs** | **Non-drug causes excluded** | **Previous hepatotoxicity data** | **Re-exposure** | **Total score** | **Causality level** |
| --- | --- | --- | --- | --- | --- | --- | --- | --- | --- |
| CQLM | +1 | +1 | +1 | 0 | +1 | 0 | 0 | 4 | Possible |
| EFJ | +2 | +1 | +1 | 0 | +1 | 0 | 0 | 5 | Possible |
| TSG | +2 | +1 | +1 | 0 | +1 | 0 | 0 | 5 | Possible |
| GSL | +2 | +3 | +1 | +1 | +2 | +1 | 0 | 9 | Highly probable |
| RGL | 0 | 0 | +1 | -1 | -2 | 0 | 0 | -3 | Unlikely |

**Table S2.** Elemental profiling of the weight-loss product (GSL sample) by ICP–OES.

Concentrations of magnesium (Mg), zinc (Zn), potassium (K), chromium (Cr), aluminum (Al), and lead (Pb) are presented. Values are expressed as ppm or % (w/w). Observed concentrations include Zn (16–17 ppm), K (2.6–2.9% w/w), Mg (~0.17% w/w), and Al (~300 ppm, undeclared). Values below detection limits are indicated as <LOD.


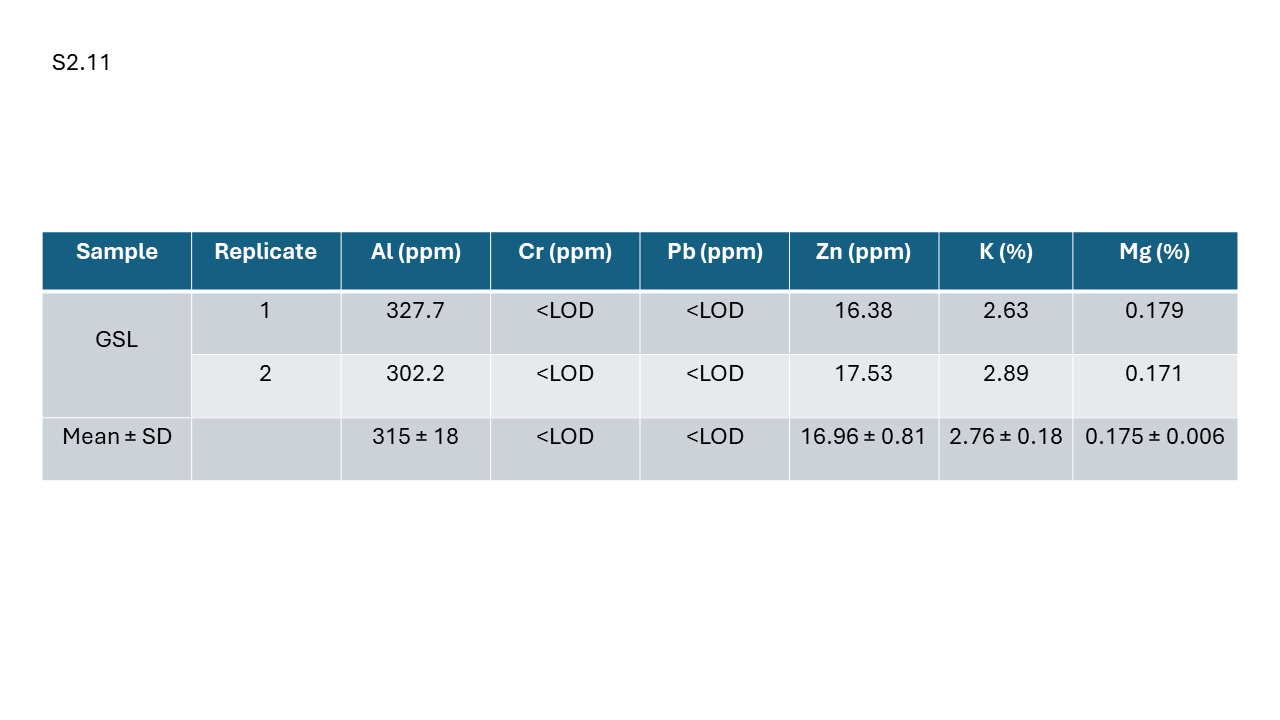


**Table S3.** Toxicity screening of aqueous extracts of herbal materials and industrialized products marketed as herbal dietary supplements (HDS), including preparations traditionally consumed as infusions (teas), from samples associated with herb-induced liver injury (HILI). Samples were evaluated at 5% (w/v) using the *Artemia salina* lethality assay (n = 120). LC₅₀ values were estimated by Probit regression. Toxicity classification followed Meyer and Clarkson criteria: ≤100 ppm (highly toxic), 100–500 ppm (moderately toxic), 500–1000 ppm (low toxicity), and >1000 ppm (non-toxic or low bioactivity).

| **Case** | **Case ID** | **Declared ingredient(s)** | **LC₅₀ (ppm)* n = 120** | **Toxicological classification** |
| --- | --- | --- | --- | --- |
| **CQLM** | CQLM1 | Cow’s foot *(Bauhinia* spp.) | 3944.07 | Non-toxic |
|  | CQLM2 | Hibiscus *(Hibiscus sabdariffa)* | 48.79 | Highly toxic |
|  | CQLM3 | Cinnamon *(Cinnamomum* spp.) | 655.13 | Low toxicity |
|  | MCQLM | CQLM-1 + CQLM-2 + CQLM-3 (3:2:1) | 313.84 | Moderately toxic |
| **EFJ** | EFJ1 | Turmeric oil with collagen - HDS | 275.19 | Moderately toxic |
|  | EFJ2 | Turmeric reference *(Curcuma longa)* | 956.45 | Low toxicity |
| **TSG** | TSG1 | Dandelion (*Taraxacum officinale*) | 1081.56 | Non-toxic |
|  | TSG2 | Horsetail (*Equisetum arvense*) | 2751.86 | Non-toxic |
|  | TSG3 | Rosemary (*Rosmarinus officinalis*) | 1924.61 | Non-toxic |
|  | MTSG | TSG-1 + TSG-2 + TSG-3 (3:2:1) | 1454.29 | Non-toxic |
| **GSL** | GSL | Weight-loss -HDS | 120.78 | Moderately toxic |
| **RGL** | RGL | Moringa (*Moringa oleifera*) | 1583.26 | Non-toxic |

**Figures**

**Figure S1.** GC–MS chromatogram of the commercial formulation “Curcumine” (EFJ-1 sample).

(A) Total ion chromatogram showing the overall chemical profile and major peaks, highlighting a low-abundance region associated with curcuma-derived metabolites (turmerone and curlone), detected at trace levels.

(B) Peak list (1–25) with retention times (RT) and relative peak areas (%). Turmerone (peak 3) and curlone (peak 4) were present at trace levels.


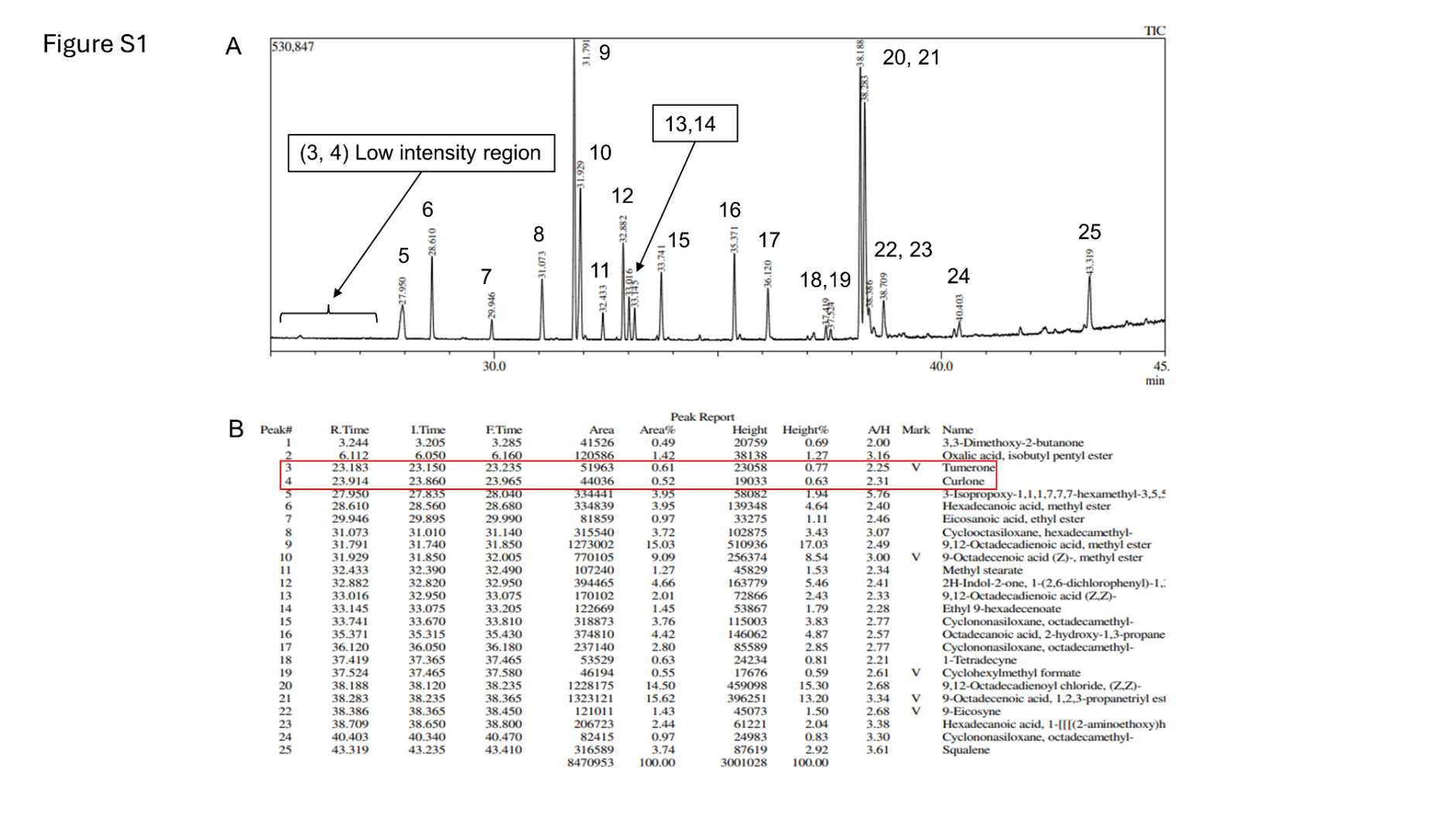


**Figure S2.** Chromatographic identification by GC-MS of the chemomarker tumerone (EFJ-1 sample).

(A) Ion fragmentation pattern of tumerone (peak 3), showing the characteristic ion and their relative intensities.

(B) Comparison of the turmerone mass spectrum with the NIST14 database, demonstrating correspondence between the experimental peaks and reference library patterns.


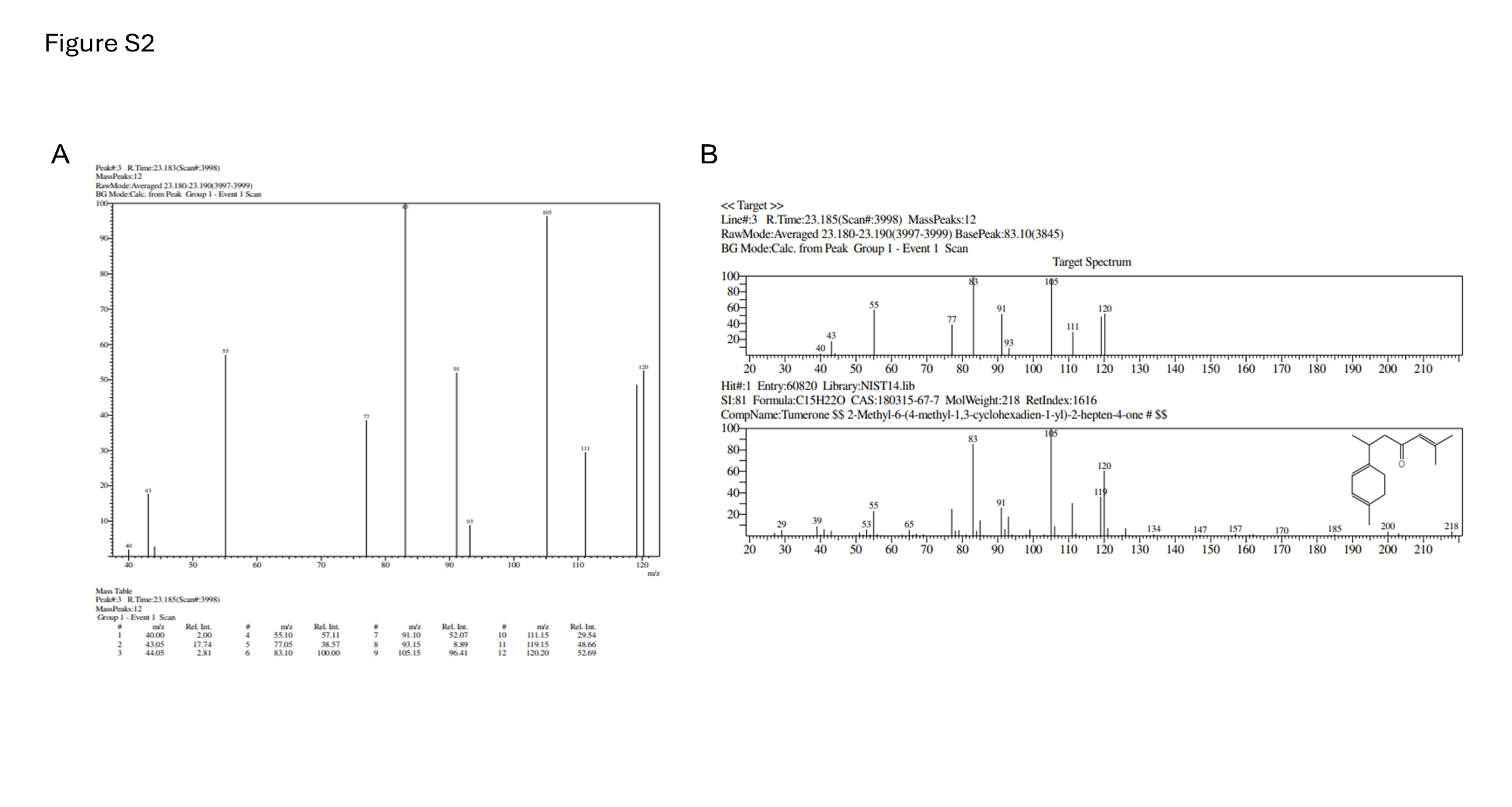


**Figure S3**. Chromatographic identification by GC-MS of the chemomarker curlone (EFJ-1 sample).

(A) Ion fragmentation pattern of curlone (peak 4), showing the characteristic ion and their relative intensities.

(B) Comparison of the curlone mass spectrum with the NIST14 database, demonstrating correspondence between the experimental peaks and reference library patterns.


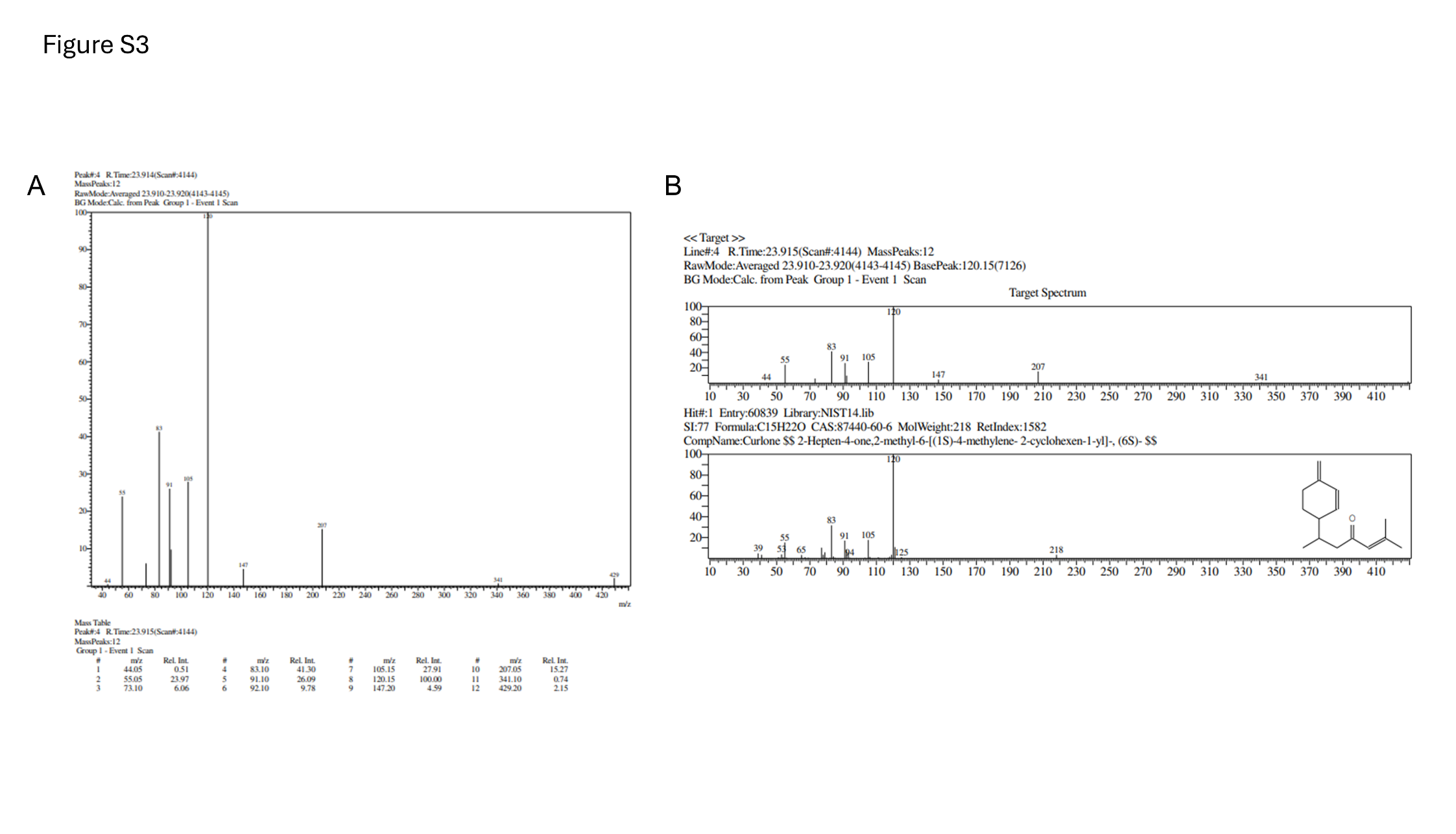


**Figure S4.** GC–MS chromatogram of the weight-loss product (GSL sample).

(A) Full chromatographic profile. Active compounds accounted for >80% of the total signal, indicating enrichment of undeclared substances relative to inert excipients. As GC–MS is semi-quantitative, amounts are approximate and require confirmation by complementary methods.

(B) Peak list (1–10) with retention times (RT) and relative peak areas (%). Major components include sibutramine (RT 25.6 min; 75.81%), fluoxetine (26.1 min; 2.64%), N-acetylfluoxetine (33.4 min; 3.89%), diazepam (36.5 min; 2.01%), bisacodyl (42.5 min; 4.16%), and bupropion

**
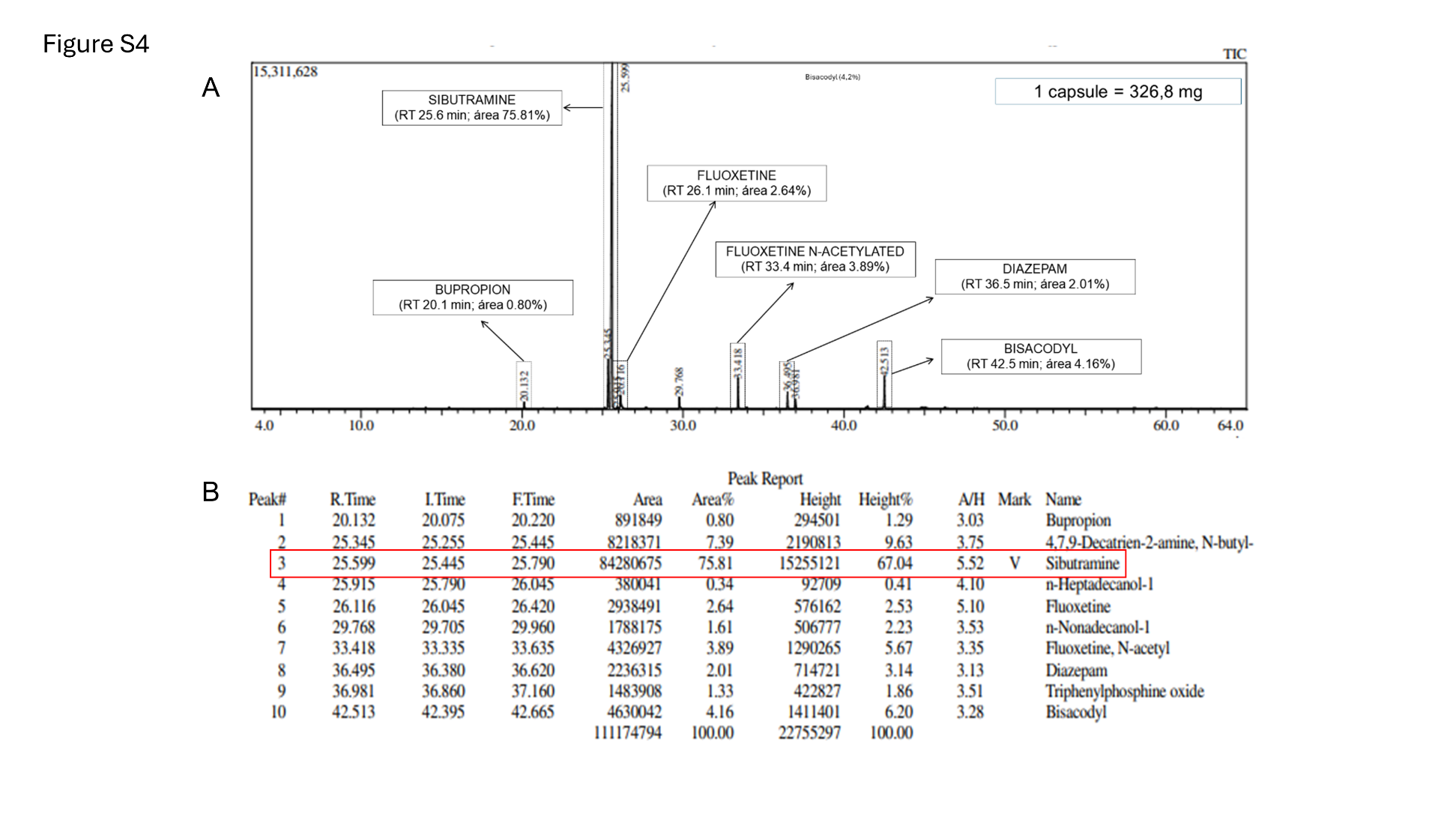
**(20.1 min; 0.80%).

**Figure S5**. Chromatographic identification by GC–MS of the major adulterant (sibutramine) in the weight-loss product (GSL sample).
(A) Ion fragmentation pattern of sibutramine (major peak), showing the characteristic ions and their relative intensities.

(B) Comparison of the sibutramine mass spectrum with the NIST14 database, demonstrating strong correspondence between the experimental spectrum and reference library patterns, supporting compound identification.


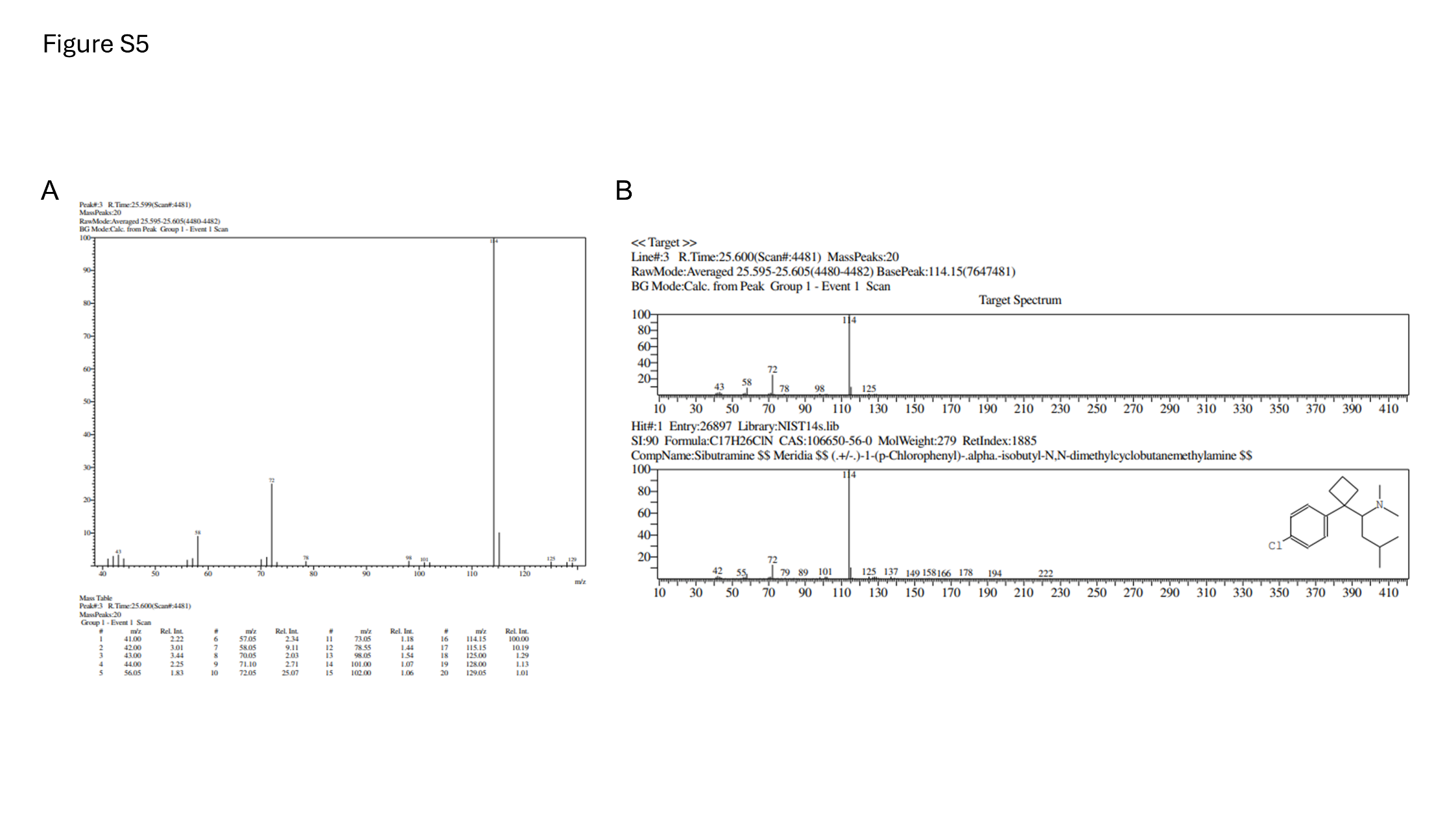


**Figure S6.** Gallic acid calibration curve obtained by UV–Vis spectrophotometry (y = 0.0044x + 0.0863; R² = 0.9975). The model was used to quantify total phenolic content, expressed as gallic acid equivalents (GAE) per 100 g of sample.


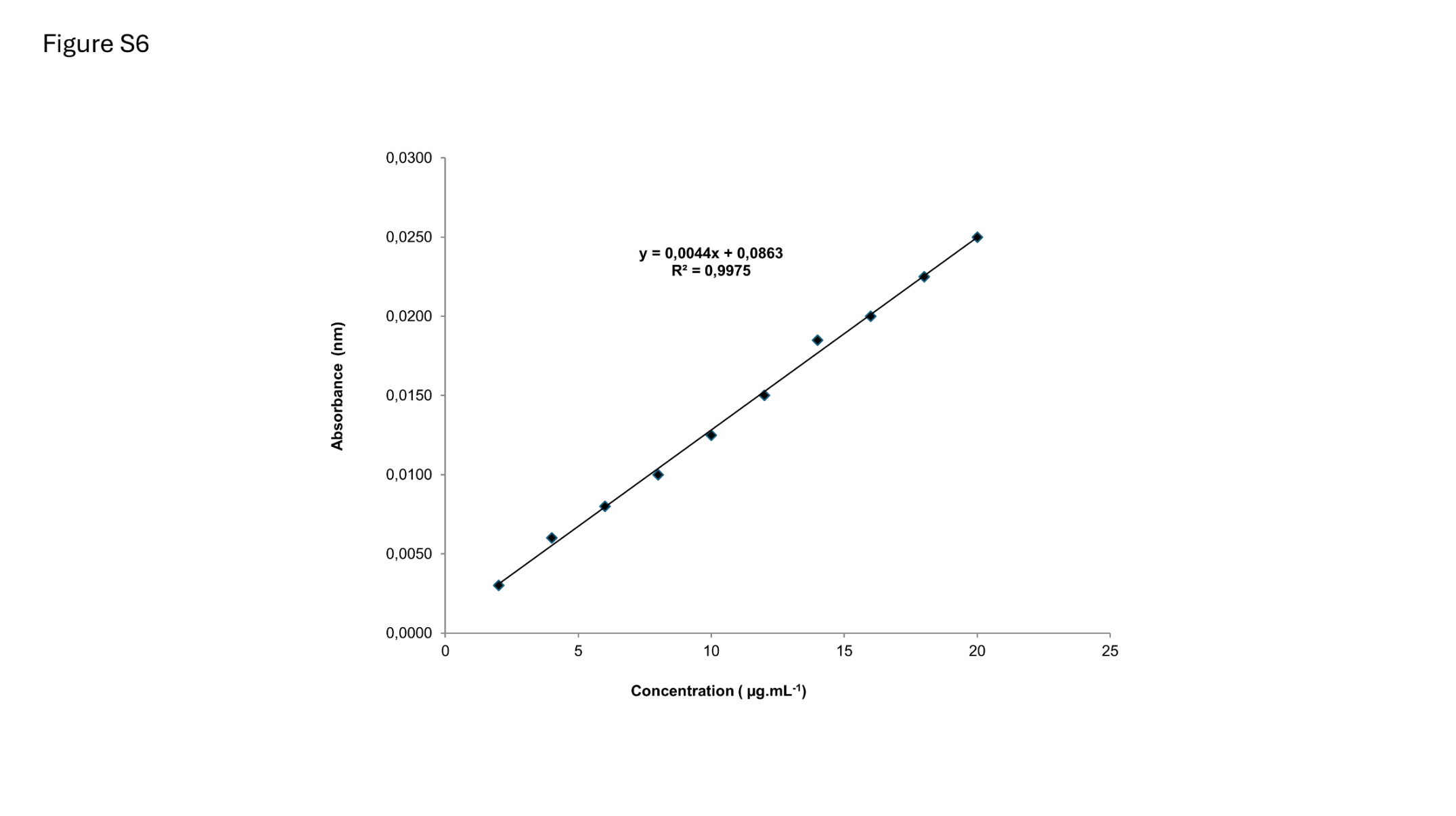


**Figure S7.** Total phenolic content of herbal materials collected from patients and prepared as hydroethanolic extracts (10% w/v). Two industrialized products marketed as herbal and dietary supplement were included in the analysis (EFJ-1 and GSL samples). The EFJ-1 sample was compared with a reference turmeric extract (EFJ-2) to assess phytochemical similarity. All samples were prepared and analyzed under identical experimental conditions. Total phenolic content was determined by UV–Vis spectrophotometry using a gallic acid calibration curve and expressed as gallic acid equivalents (mg GAE/100 g). Butylated hydroxytoluene (BHT) and rutin were included as reference compounds.


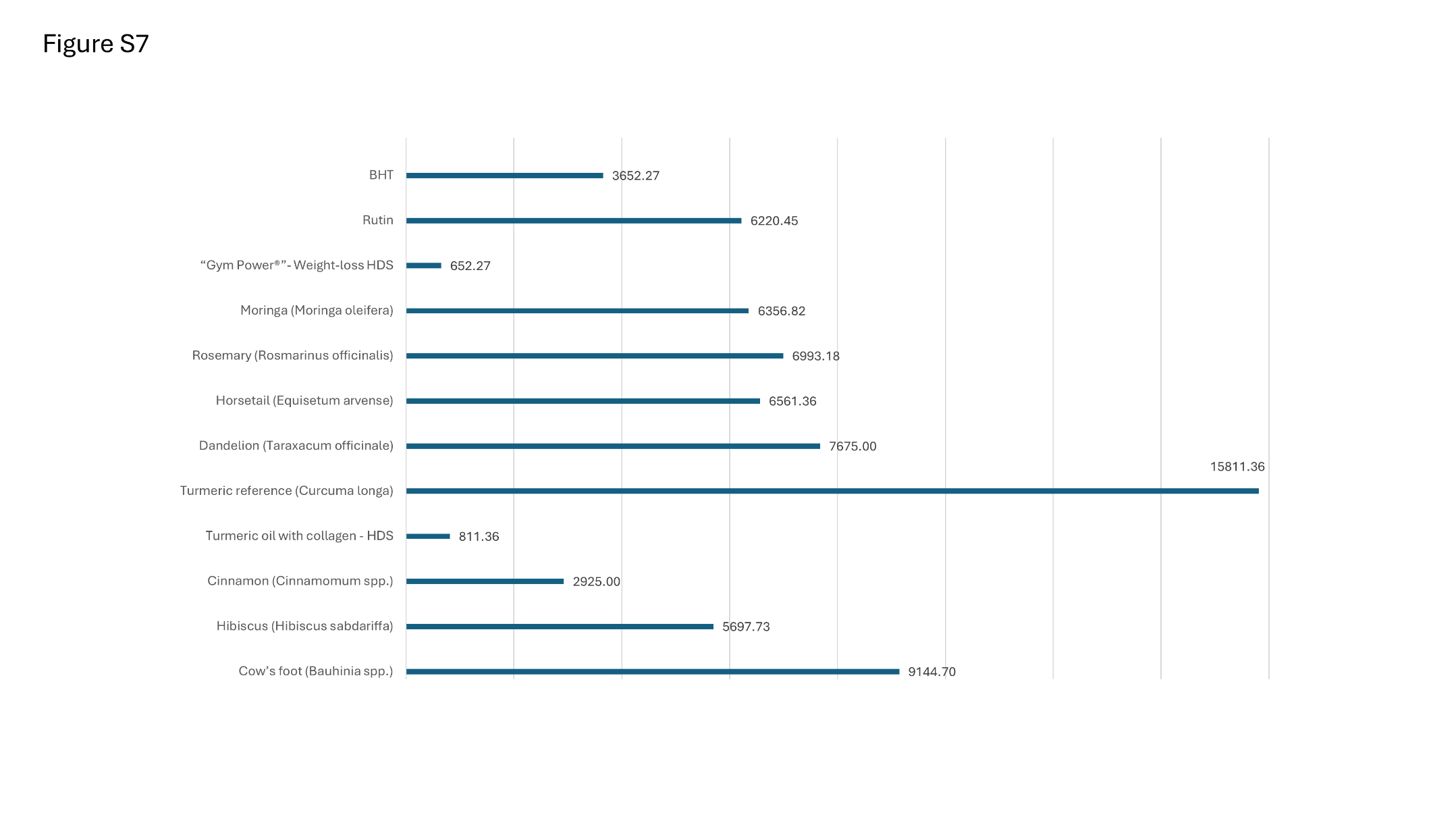


**Figure S8.** Quercetin calibration curve obtained by UV–Vis spectrophotometry (y = 0.0032x + 0.0012; R² = 0.9982). The model was used to quantify total flavonoid content, expressed as quercetin equivalents (mg QE/g of sample).


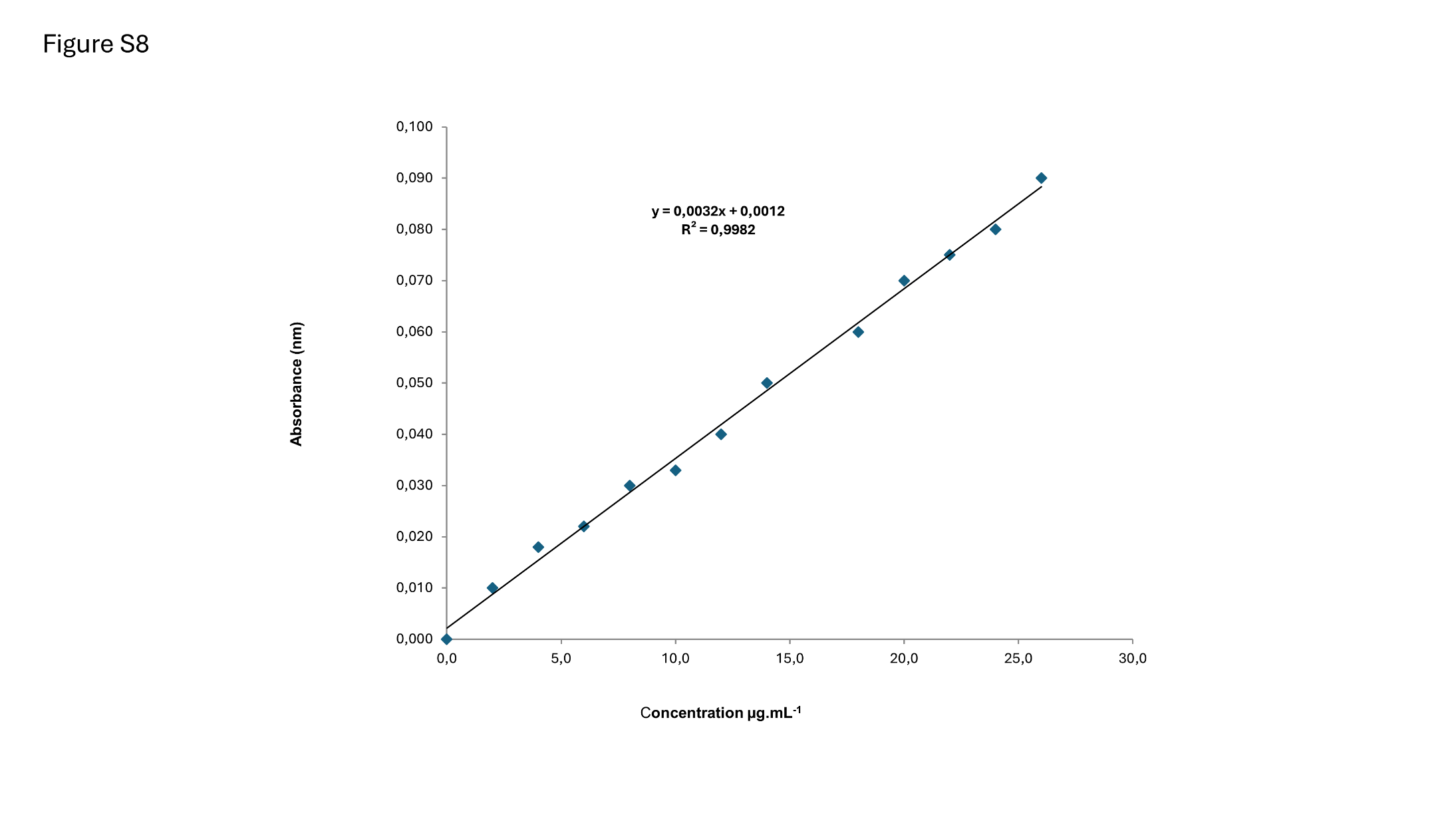


**Figure S9.** Total flavonoid content of herbal materials collected from patients and prepared as hydroethanolic extracts (10% w/v). Two industrialized products marketed as herbal and dietary supplements were included in the analysis (EFJ-1, turmeric oil with collagen; and GSL, weight-loss product). The EFJ-1 sample was compared with a reference turmeric extract (EFJ-2) to assess phytochemical similarity. Marked differences in flavonoid content were observed between commercial formulations and traditional herbal extracts prepared for infusion (tea). Total flavonoid content was determined by UV–Vis spectrophotometry using a quercetin calibration curve and expressed as quercetin equivalents (mg QE/g of sample). Rutin was included as a reference compound.


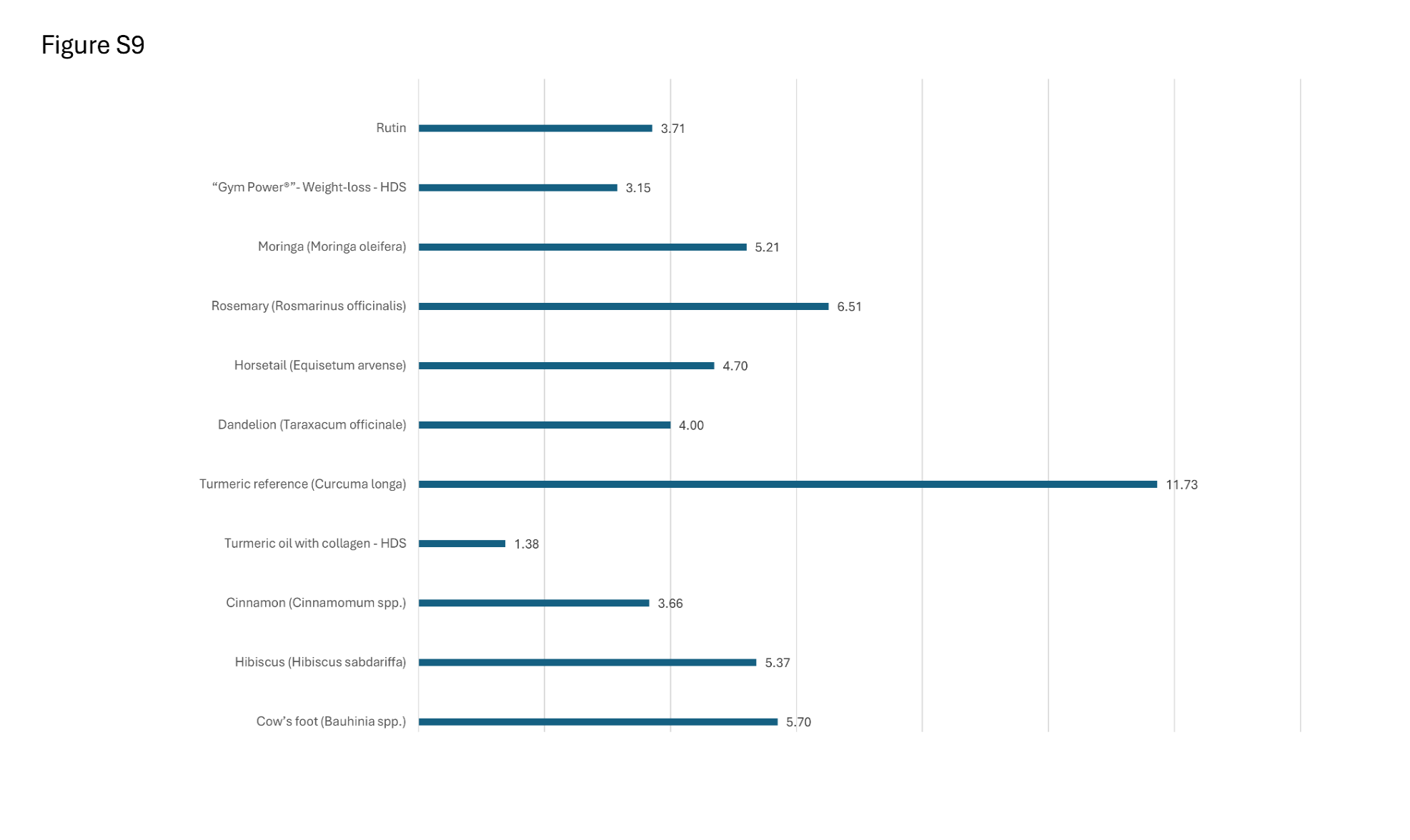


**Figure S10.** Antioxidant activity of herbal materials and industrialized products marketed as herbal and dietary supplements (EFJ-1, turmeric oil with collagen; GSL, weight-loss product), determined 2,2-diphenyl-1-picrylhydrazyl (DPPH) radical scavenging assay. Results are expressed as percentage of DPPH inhibition (%) or antioxidant capacity, as indicated.


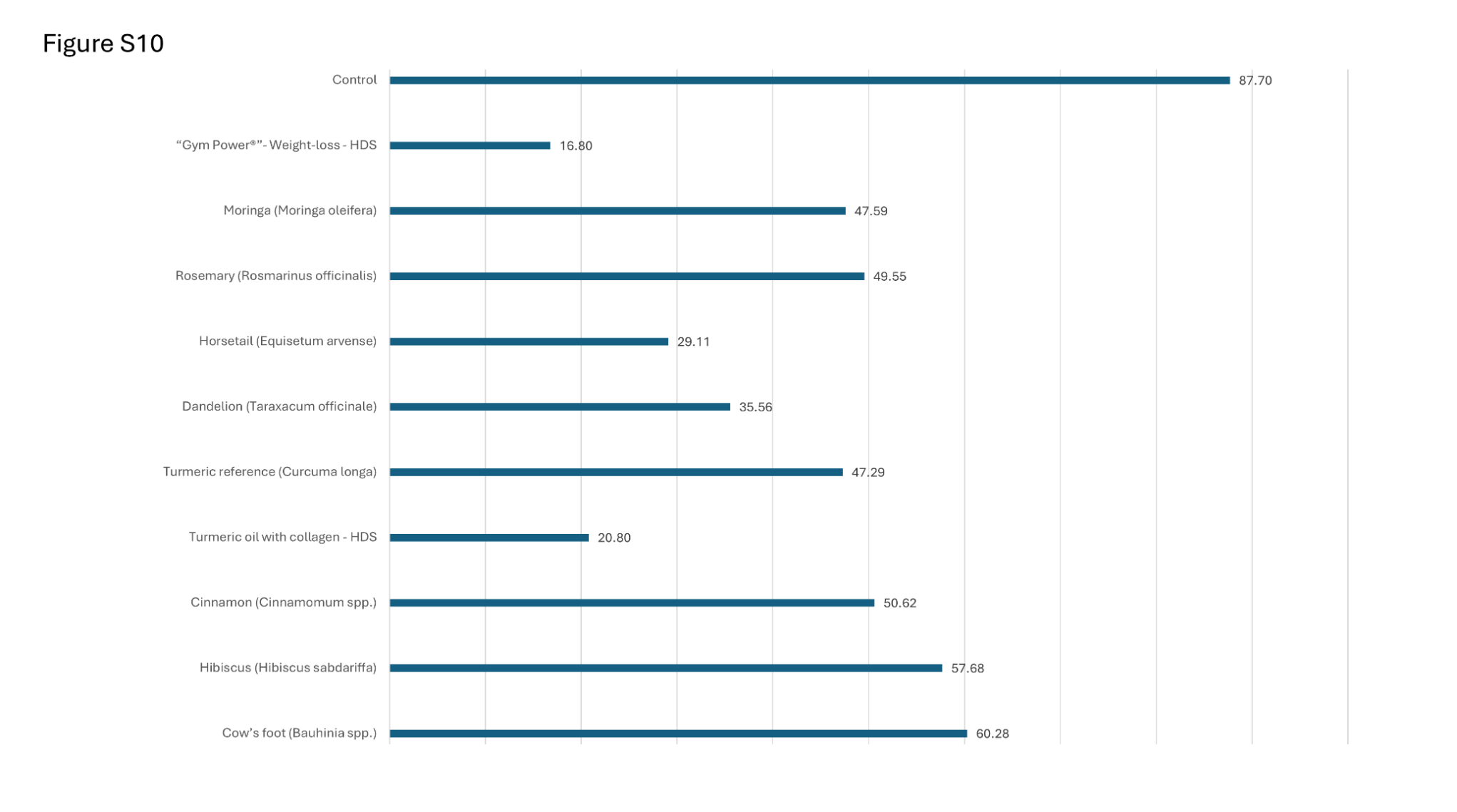


**Figure S11.** Ferric reducing antioxidant power (FRAP) of herbal materials, traditional herbal infusions (teas), and commercially manufactured products marketed as herbal formulations. Antioxidant capacity was determined using the FRAP assay and quantified based on a ferrous sulfate calibration curve (y = 0.0002x + 0.086; R² = 0.9984).


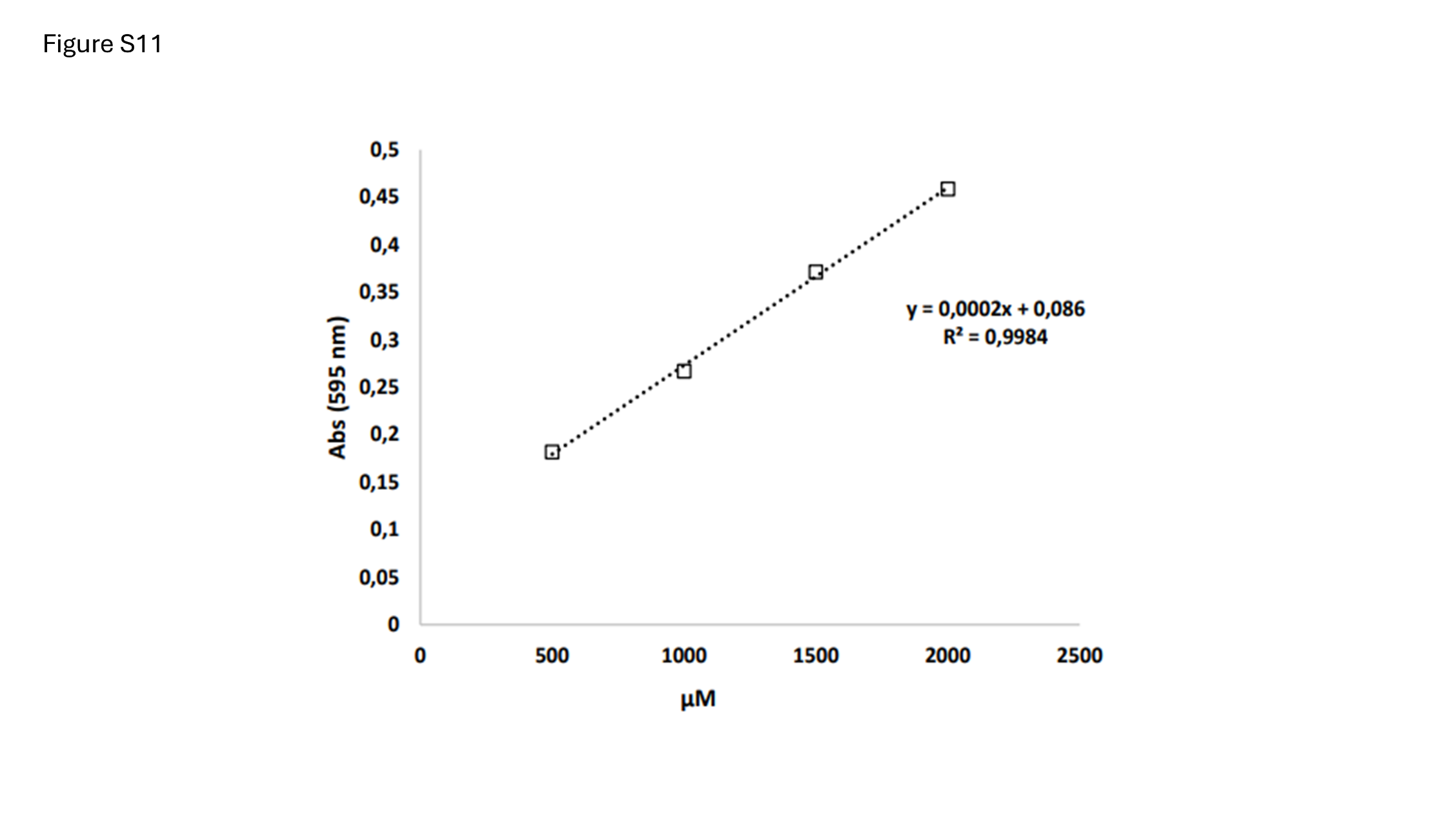


**Figure S12.** Ferric reducing antioxidant power (FRAP) of herbal materials and commercially manufactured products marketed as herbal formulations (EFJ-1 and GSL). Samples were prepared as hydroethanolic extracts at 10% (w/v) prior to analysis. Results are expressed as ferric reducing antioxidant power based on Fe³⁺–TPTZ reduction.


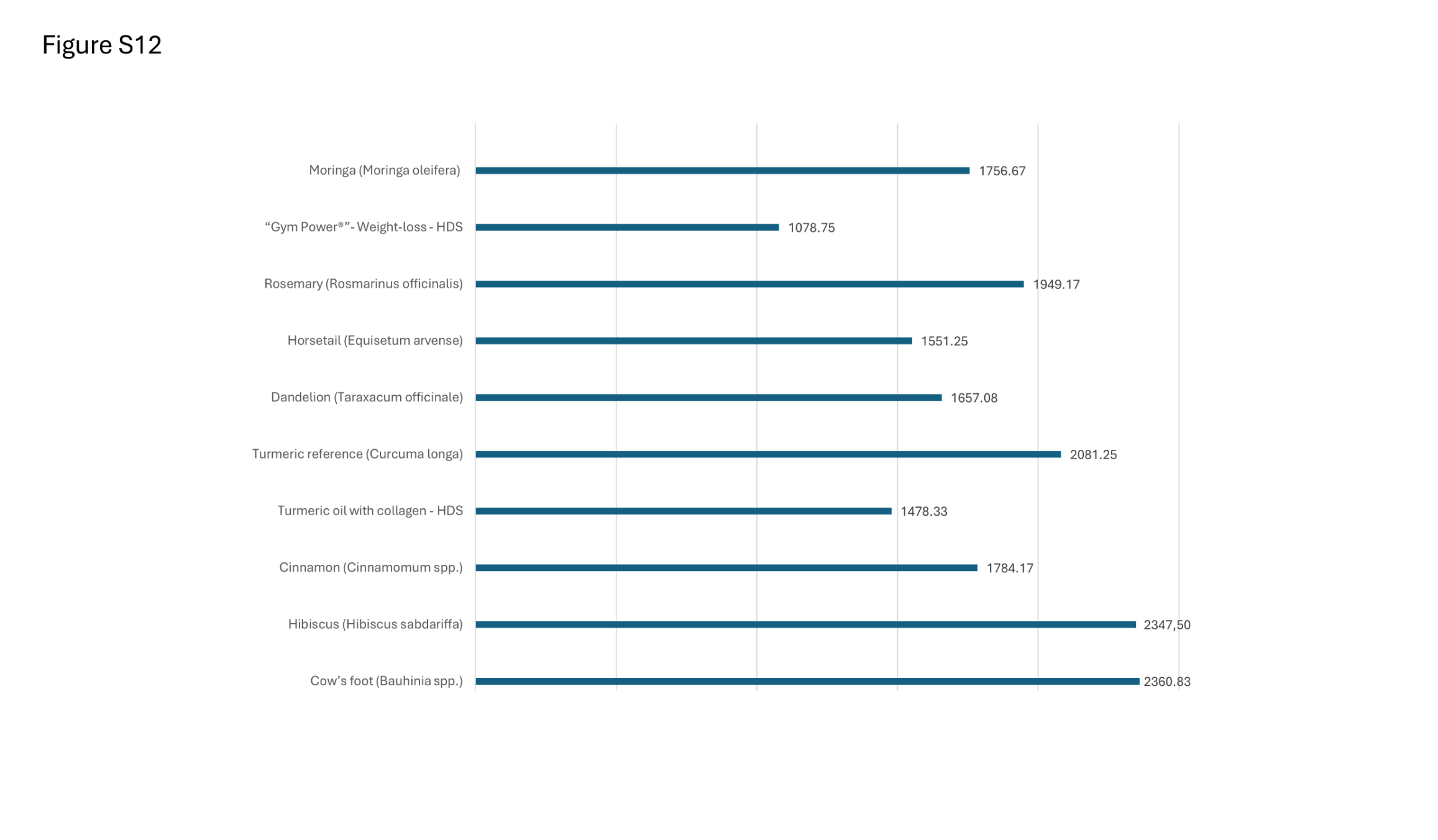


**Figure S13.** Comparative LC₅₀ values (ppm) of aqueous herbal extracts and industrialized products marketed as herbal formulations (EFJ-1, turmeric oil with collagen; GSL, weight-loss product), evaluated at 5% (w/v) using the *Artemia salina* lethality bioassay (n = 120 nauplii). LC₅₀ values were estimated by Probit regression analysis. Toxicity classification followed Meyer and Clarkson criteria: ≤100 ppm, highly toxic; 100–500 ppm, moderately toxic; 500–1000 ppm, low toxicity; >1000 ppm, non-toxic or low bioactivity.


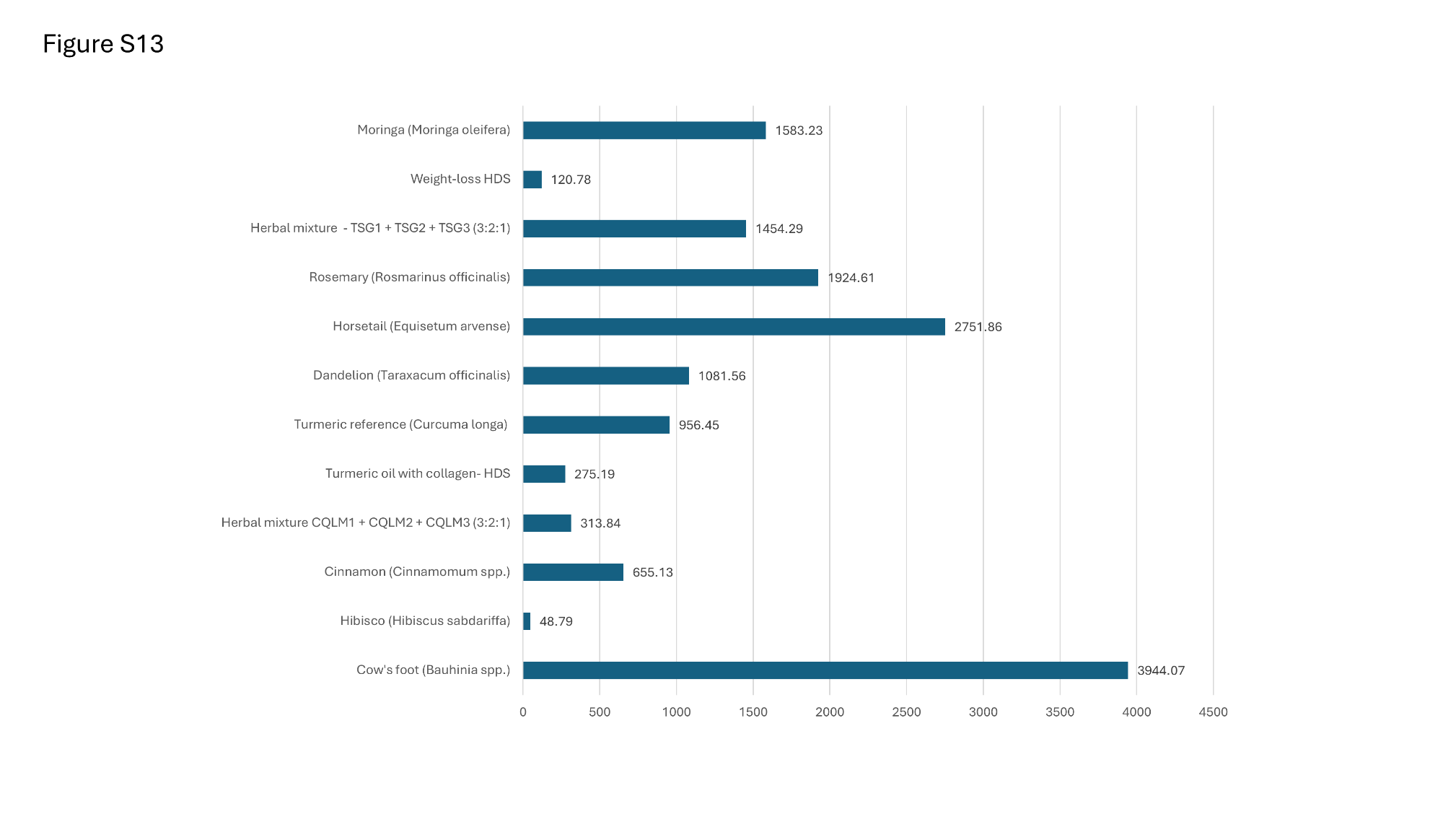


**Figure S14.** Photographic documentation of two commercially manufactured products marketed as herbal and dietary supplements and suspected of hepatotoxicity. Panels A and B show the EFJ-1 sample (“Curcumine” – turmeric oil with collagen), including front (A) and back (B) label views with manufacturer information. Panels C and D show the GSL sample (“Gym Power Gold”), including front (C) and back (D) label views. Panel E shows the accompanying package insert containing instructions for use and consumer information.


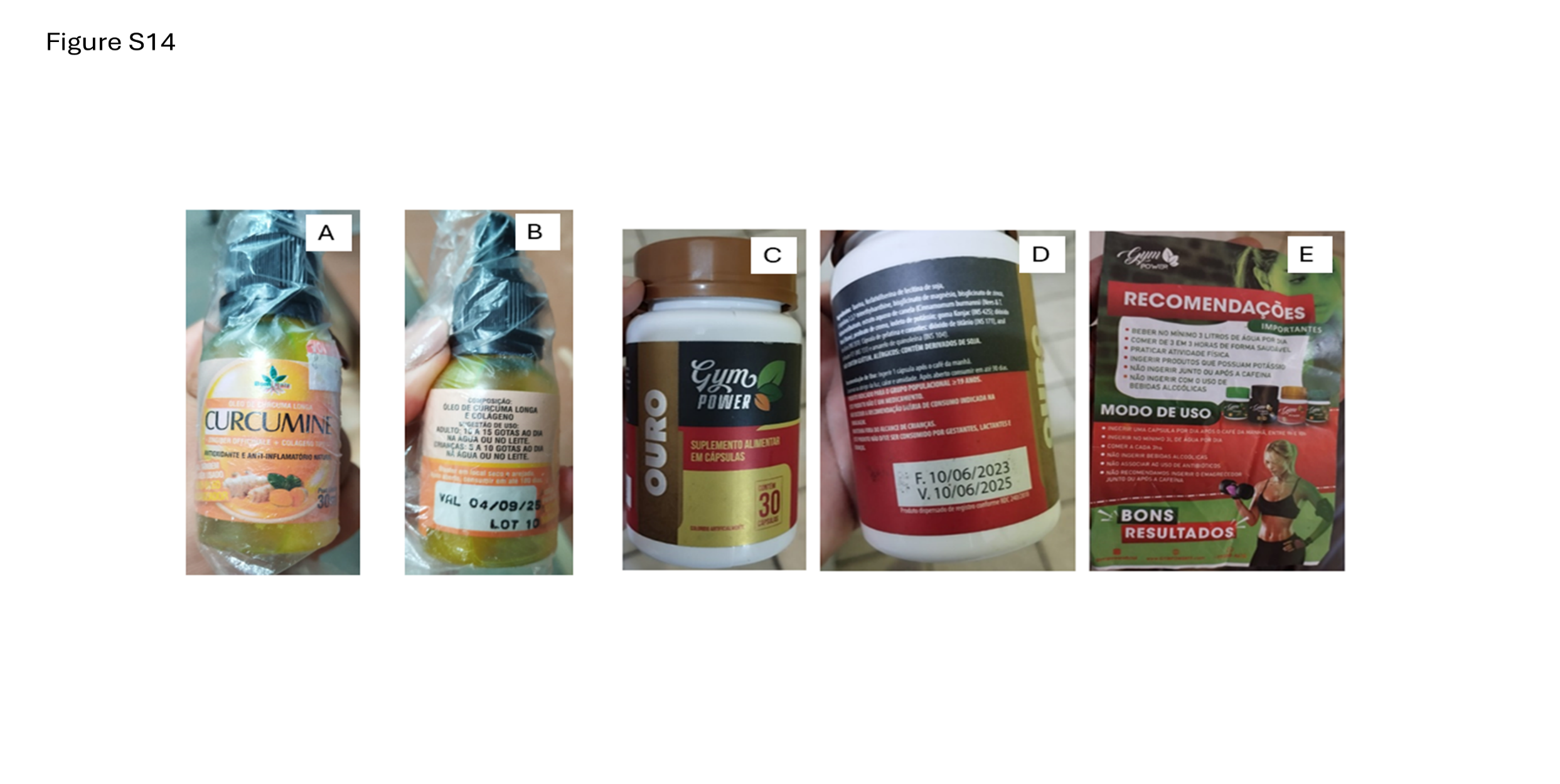


**Methods**

**RUCAM Causality Assessment**

Causality between exposure to herbal materials and commercial formulations and the occurrence of liver injury was assessed using the updated RUCAM (Roussel Uclaf Causality Assessment Method). Each case was independently evaluated by two investigators based on clinical history, laboratory data, and the systematic exclusion of alternative etiologies.

**The following domains were scored according to the updated RUCAM criteria:**1. Time to onset from supplement intake
2. Course of ALT after cessation
3. Risk factors
4. Concomitant medications
5. Exclusion of alternative causes
6. Known hepatotoxicity of the product
7. Response to re-exposure when available

**Final scores were interpreted as:**
≤0 excluded, 1–2 unlikely, 3–5 possible, 6–8 probable, and ≥9 highly probable.

The individual domain scores obtained using the updated RUCAM are summarized in Supplementary Results (Section S4: Table S1).

**Chemical and Phytochemical Analysis of Herbal and Commercial Formulations**

**GC–MS Instrumentation and Analytical Conditions**

Chemical characterization of commercial formulations (EFJ-1 and GSL) was performed by gas chromatography–mass spectrometry (GC–MS) using a Shimadzu GC-2010 gas chromatograph coupled to a GCMS-QP2020 mass spectrometer. The chromatographic analyses were conducted at the facilities of the Analytical Center of the University of São Paulo (USP), located in São Paulo, Brazil.

Samples were introduced using split injection mode with injector temperature maintained at 280 °C. Helium was employed as carrier gas under linear velocity control (~51.6 cm s⁻¹), with column flow approximately 2.0 mL min⁻¹, total flow ~17.4 mL min⁻¹, inlet pressure ~17.9 psi, and split ratio approximately 6:1. Purge flow was set at 3.0 mL min⁻¹, with equilibrium time of 1.0 min.

The oven temperature program started at 60 °C and increased to 280 °C, ensuring elution of semi-volatile and high-boiling lipophilic constituents (total run ~65 min).

Mass spectrometric detection was conducted in electron ionization mode with ion source and interface temperatures at 280 °C. Data acquisition was performed in full scan mode (m/z 37–660) with solvent delay of 3.0 min, scan speed ~2500 amu s⁻¹, and event time 0.30 s.

**Compound identification was based on:**

- Spectral library matching
- Retention time consistency
- Fragmentation pattern interpretation

Peak areas were used for semi-quantitative estimation. GC–MS methodology is appropriate for low-molecular-weight compounds but does not reliably detect intact proteins or peptides. GC–MS analysis and compound identification are provided in the Supplementary Results (Section S5; Figures S1–S5).

**ICP–OES Instrumentation and Elemental Analysis**

Elemental profiling (GSL sample) was performed by inductively coupled plasma optical emission spectrometry (ICP-OES) using an Agilent 5800 optical emission spectrometer.

Samples underwent closed-vessel microwave digestion using concentrated nitric acid and 30% hydrogen peroxide at approximately 200 °C to ensure complete mineral extraction and organic matrix decomposition.

Quantification employed certified multi-element calibration standards, with all measurements performed in duplicate for mean value calculation. Analytical performance included calibration linearity verification, blank controls, and detection limit assessment (~0.01 mg L⁻¹ after digestion/dilution). Results were expressed as mass fraction (%) or parts per million (ppm) and are presented in Supplementary Results (Section S5; Table S2). Declared minerals (Mg, Zn, K, Cr) and potential contaminants (e.g., Al, Pb) were evaluated to assess label concordance and possible toxicological relevance.

The elemental analyses were conducted at the facilities of the Analytical Center of the University of São Paulo (USP), located in São Paulo, Brazil.

**UV–Vis Phytochemical Characterization and Antioxidant Activity Assessment**

A comparative phytochemical approach was adopted, encompassing commercial formulations and aqueous extracts prepared to simulate traditional herbal infusions (teas). The aim was to assess chemical composition (authenticity) and the occurrence of herbal secondary metabolites, with focus on bioactive potential.

UV–Vis-based assays, including total phenolic content, flavonoid content, and antioxidant activity (DPPH and FRAP), were performed as described, with calibration curves and analytical results presented in Supplementary Results (Section S5; Figures S6–S12.

**Sample preparation and assays were standardized:**

- Extraction procedures, solvent compositions, and volumes were consistent across all samples. Approximately 0.1 g of sample was extracted, and appropriate dilutions (typically 1:10) were applied prior to spectrophotometric analysis;
- Microplate measurements were performed using a BioTek 800 TS ELISA reader at the Natural Products Research Laboratory, Faculty of Pharmacy, UFBA.
- Calibration curves with certified reference standards were constructed under identical conditions;

**Assays included:**

- Total Phenolic Content (Folin–Ciocalteu method) – adapted for 96-well plates, with calibration curve linearity (R^2^ = 0.9975).
- Total Flavonoid Content (AlCl₃ complexation) - microplate assay format, with calibration curve linearity (R^2^=0.9982).
- 2,2-diphenyl-1-picrylhydrazyl (DPPH) radical scavenging assay – microplate format with triplicate measurements at multiple concentrations.
- Ferric Reducing Antioxidant Power (FRAP) – based on Fe³⁺–TPTZ reduction, with calibration curve (R^2^ = 0.9984).

Spectrophotometric measurements were performed using a microplate ELISA reader (BioTek 800 TS) available at the Natural Products Research Laboratory, Faculty of Pharmacy, Federal University of Bahia (UFBA).

**Total Phenolic Content**

Total phenolic compounds were determined using the Folin–Ciocalteu colorimetric method adapted for 96-well microplates. Aliquots (20 µL) of hydroethanolic extracts (10% w/v prepared in 80% ethanol) were mixed with 100 µL Folin–Ciocalteu reagent followed by 75 µL sodium carbonate solution. Blank reactions were prepared using extraction solvent instead of sample. After incubation for 30 min at room temperature protected from light, absorbance was measured at 765 nm. The results were expressed as milligrams of gallic acid equivalents per gram of sample (mg GAE/g) based on calibration with gallic acid standard.

**Total Flavonoid Content**

Flavonoid content was determined by aluminum chloride complexation. Aliquots (100 µL) of extracts or quercetin standards were combined with 100 µL AlCl₃ solution and 100 µL methanol in 96-well plates. After 30 min incubation protected from light, absorbance was recorded at 415 nm. The results were expressed as milligrams of quercetin equivalents per gram of extract.

**DPPH Radical Scavenging Assay**

Antioxidant activity was evaluated using the DPPH radical scavenging assay adapted to microplate format. Extracts were tested in triplicate at concentrations of 1.0, 0.5, and 0.25 mg·mL⁻¹. For each assay, 50 µL sample was added to 250 µL methanolic DPPH solution (200 µmol·L⁻¹). Butylated hydroxytoluene (BHT) was used as positive control.

Reactions were conducted at 25 ± 1 °C protected from light. Absorbance was monitored at 517 nm after 15, 30, 45, 60, and 90 min; the 45-min reading was used for comparative purposes. Antioxidant activity was expressed as percentage inhibition (%I), and IC₅₀ values were obtained by regression analysis. Results represent mean ± standard deviation of three independent determinations.

**Ferric Reducing Antioxidant Power (FRAP) Assay**

Total antioxidant capacity was assessed by the FRAP method based on reduction of the Fe³⁺–TPTZ complex to Fe²⁺–TPTZ. The reagent consisted of acetate buffer (300 mM, pH 3.6), TPTZ solution (10 mM in 40 mM HCl), and ferric chloride solution (20 mM) mixed in a 10:1:1 ratio.

For analysis, 10 µL extract, 25 µL distilled water, and 265 µL FRAP reagent were combined (final volume 300 µL). After 30 min incubation protected from light, absorbance was measured in a microplate reader. Results were expressed as micromoles ferrous sulfate equivalents per gram of dry sample (µM FeSO₄/g), based on calibration curves (y = 0.0002x + 0.086; R² = 0.9984).

**In vitro toxicological screening using *Artemia salina***

In vitro toxicity screening was performed using the *Artemia salina* bioassay, a widely applied preliminary method for estimating overall biological toxicity. Samples with LC50 values below 1000 ppm were considered biologically active and therefore selected for subsequent cytotoxicity testing in HepG2 liver cells.

Cysts were incubated in artificial saline solution (0.35% w/v sea salt) under continuous aeration and constant illumination for approximately 48 hours, until nauplii hatched. Herbal products suspected of being associated with liver injury (HILI) were prepared as aqueous extracts obtained by 5% (w/v) infusion, simulating traditional consumption practices, and diluted to four concentrations (4.0, 2.0, 1.0, and 0.5 mg/mL). Groups of ten viable nauplii were transferred to test tubes containing the experimental solutions, in triplicate, and kept at room temperature for 24 hours (n = 120 nauplii).

Toxicity screening results obtained using the *Artemia salina* lethality assay are presented in Supplementary Results (Section S6; Figure S13 and Table S3).
